# Loss of MeCP2 in the rat models regression, impaired sociability and transcriptional deficits of Rett syndrome

**DOI:** 10.1093/hmg/ddw178

**Published:** 2016-06-30

**Authors:** Surabi Veeraragavan, Ying-Wooi Wan, Daniel R. Connolly, Shannon M. Hamilton, Christopher S. Ward, Sirena Soriano, Meagan R. Pitcher, Christopher M. McGraw, Sharon G. Huang, Jennie R. Green, Lisa A. Yuva, Agnes J. Liang, Jeffrey L. Neul, Dag H. Yasui, Janine M. LaSalle, Zhandong Liu, Richard Paylor, Rodney C. Samaco

**Affiliations:** 1 Department of Molecular and Human Genetics; 2 Department of Pediatrics, Section of Neurology; 3 Program in Translational Biology and Molecular Medicine; 4 Medical Scientist Training Program, Baylor College of Medicine, Houston, TX, USA; 5 Jan and Dan Duncan Neurological Research Institute, Texas Children’s Hospital, Houston, TX, USA; 6 Rowe Program in Human Genetics, University of California Davis, Davis, CA, USA

## Abstract

Mouse models of the transcriptional modulator Methyl-CpG-Binding Protein 2 (MeCP2) have advanced our understanding of Rett syndrome (RTT). RTT is a ‘prototypical’ neurodevelopmental disorder with many clinical features overlapping with other intellectual and developmental disabilities (IDD). Therapeutic interventions for RTT may therefore have broader applications. However, the reliance on the laboratory mouse to identify viable therapies for the human condition may present challenges in translating findings from the bench to the clinic. In addition, the need to identify outcome measures in well-chosen animal models is critical for preclinical trials. Here, we report that a novel *Mecp2* rat model displays high face validity for modelling psychomotor regression of a learned skill, a deficit that has not been shown in *Mecp2* mice. Juvenile play, a behavioural feature that is uniquely present in rats and not mice, is also impaired in female *Mecp2* rats. Finally, we demonstrate that evaluating the molecular consequences of the loss of MeCP2 in both mouse and rat may result in higher predictive validity with respect to transcriptional changes in the human RTT brain. These data underscore the similarities and differences caused by the loss of MeCP2 among divergent rodent species which may have important implications for the treatment of individuals with disease-causing *MECP2* mutations. Taken together, these findings demonstrate that the *Mecp2* rat model is a complementary tool with unique features for the study of RTT and highlight the potential benefit of cross-species analyses in identifying potential disease-relevant preclinical outcome measures.

## Introduction

Rett syndrome (RTT, MIM 312750) is an X-linked neurodevelopmental disorder caused by mutations in *MECP2* (1). Typical RTT is characterized by a period of apparently normal development followed by the loss of acquired skills and a striking pattern of disease onset and progression (2,3). In addition, disease-causing *MECP2* mutations and genomic abnormalities have been reported in a wide range of disorders such as autism (4–6), schizophrenia (7–9), intellectual disability (10–12), developmental delay, obsessive compulsive disorder, attention deficit-hyperactivity disorder (13), and *MECP2* duplication syndrome (14–16), and have also been reported in individuals with mild cognitive and behavioural impairments (17–24). Thus, the true impact of *MECP2* mutations may be wider than is often appreciated, as suggested by work identifying common variants of *MECP2* as potential risk factors for autism and autism spectrum disorders (ASD) (25). Because of the overlapping disease features of RTT with other intellectual disabilities and autism spectrum disorders (IDD/ASD), therapeutic interventions that may help RTT phenotypes may also prove useful for the treatment of many neurodevelopmental and neurological conditions.

Recent studies in animal models have demonstrated that RTT may indeed be reversible; most notably, many features of the disorder are normalized in mouse models following re-expression of the *Mecp2* gene (26). Phenotypic deficits are also induced following deletion of the gene in adult mice, arguing that the disease is the result of functional abnormalities of neurons and/or neural circuits rather than abnormal development (27). Although many promising therapeutic leads (28–33) have emerged, the field must proceed with caution. Historically, the vast majority of experimental therapies for neurological disorders failed to translate successfully from animal models to human disease therapies for reasons such as sub-optimal animal models, poor study design, and the lack of rigorous evaluations (34–37).

Given these challenges and potential limitations of genetic mouse models (37–39), additional genetic mammalian model systems may provide powerful insight into alterations that are not always readily captured using the mouse, and importantly, may also be necessary for identifying needed preclinical measures. Rats provide an alternative rodent model and are currently the translational model system of choice for validation of therapeutic strategies (40). In addition, rats display sophisticated social and cognitive behaviours (41,42), some of which are not present in mice (43). The purpose of our study was to therefore determine the extent to which constitutive loss of MeCP2 in female rats recapitulates behavioural features of RTT. In addition, by studying the molecular consequences of loss of MeCP2 in a second mammalian rodent species, we may identify evolutionarily conserved alterations that may have better disease relevance to RTT pathogenesis.

## Results

### The *Mecp2* zinc-finger nuclease rat is a viable model for studies of MeCP2 loss-of-function

**Figure 1.**
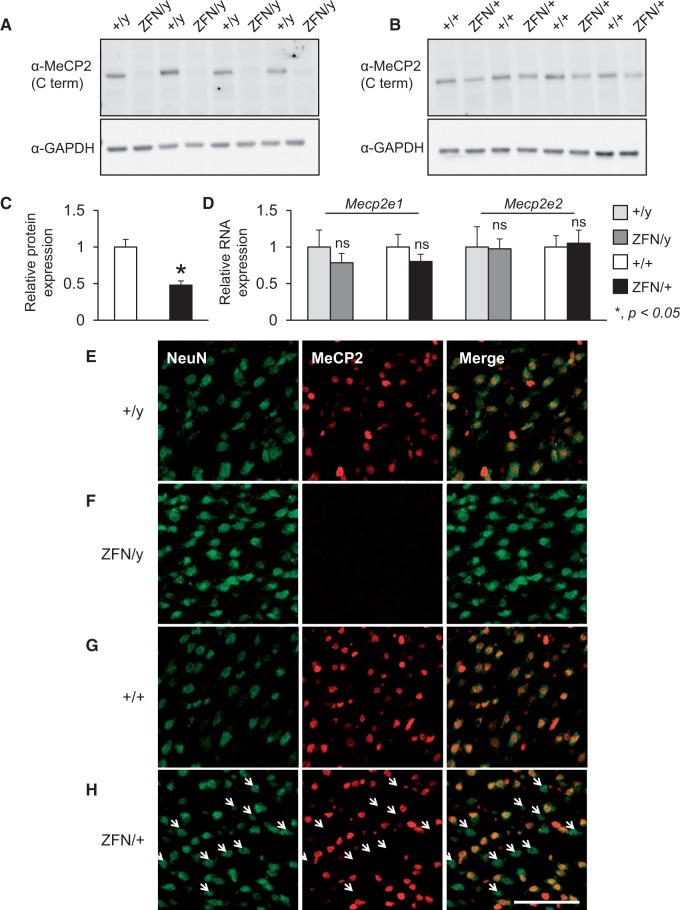
The *Mecp2* zinc-finger nuclease (ZFN) rat is a viable model to study the consequences of the loss of MeCP2 function. (**A–C**) Western blot analysis of brain tissue obtained from male *Mecp2*^ZFN/y^ (ZFN/y) and *Mecp2*^ZFN/+ ^(+/+) rats compared with corresponding male and female wild-type littermate male animals (*n =* 4 per genotype per sex). A C-terminal MeCP2 antibody does not detect MeCP2 in male *Mecp2*^ZFN/y^ rats (A). Female *Mecp2*^ZFN/+^rats display an approximate 50% reduction in MeCP2 protein levels (B, C). (**D**) ZFN targeting of the endogenous rat *Mecp2* locus does not alter *Mecp2* RNA expression levels of either the *Mecp2e1* or *Mecp2e2* isoforms in cortical tissue of male or female *Mecp2* rats as measured by quantitative real-time RT-PCR (*n =* 4 per genotype per sex). (**E–H**) Representative images of immunofluorescence staining of cortical tissue using a C-terminal MeCP2 antibody detects MeCP2 (red) in NeuN-positive cells (green) in male wild-type littermate rats (+/y) (E), but not male *Mecp2*^ZFN/y^ rats (ZFN/y) (F). Female wild-type littermate rats (+/+) show normal MeCP2 staining (G); in contrast, female *Mecp2*^ZFN/+^rats (ZFN/+) show a mosaic pattern of MeCP2 expression (H). Quantification of MeCP2 signal intensity in female *Mecp2*^ZFN/+^rats compared with female wild-type littermate rats confirms an approximate 50% reduction in MeCP2 levels, confirming observations by Western blot analysis; MeCP2 is detected in approximately half of NeuN-positive cells (Supplementary Material, Figure S3). White arrows indicate the loss of MeCP2 in NeuN-positive cells, scale bar indicates 100 µm. +/y, male wild-type littermate, +/+, female wild-type littermate; ZFN/y, *Mecp2*^ZFN/y^; ZFN/+, *Mecp2*^ZFN/+;^ *P < 0.05; ns, not significant.

*Mecp2* rats were generated using a zinc-finger nuclease (ZFN) strategy (44) that resulted in a 71 base pair deletion within exon 4 and is predicted to result in an early stop codon at amino acid 245 (Supplementary Material, Fig. S1). Western blot analysis of forebrain tissue using a C-terminal anti-MeCP2 antibody demonstrated a complete loss of full-length MeCP2 in male rats lacking *Mecp2* (*Mecp2*^ZFN/y^) and an approximate 50% reduction in MeCP2 levels in female rats lacking one copy of *Mecp2* (*Mecp2*^ZFN/+^) (Figure 1A–C; Supplementary Material, Fig. S2A, B). However, quantification of *Mecp2e1* and *Mecp2e2* RNA isoforms by QPCR showed normal expression at the transcript level (Figure 1D), suggesting that modification of the endogenous rat *Mecp2* locus resulted in post-transcriptional loss of MeCP2 protein. Alterations in protein levels were confirmed by immunofluorescence (IF) staining of cortical tissue. Male *Mecp2*^ZFN/y^ rats showed a complete absence of MeCP2 in NeuN-positive cells as expected in male rats carrying only the mutant copy of *Mecp2* (Figure 1E,F). In contrast, female *Mecp2*^ZFN/+ ^rats demonstrated a heterogeneous pattern of MeCP2 expression as expected in females carrying one mutant and one normal copy of *Mecp2* (Figure 1G,H). In addition, quantification of MeCP2 staining in cortical tissue from female *Mecp2*^ZFN/+ ^rats also showed an approximate 50% reduction in MeCP2 IF signal intensity with approximately half of NeuN-positive cells expressing MeCP2 (Supplementary Material, Fig. S3). Furthermore, although the ZFN-induced deletion is predicted to result in a truncated protein, an N-terminal anti-MeCP2 antibody did not reveal any detectable product by Western blot (Supplementary Material, Fig. S2C) or IF staining (Supplementary Material, Fig. S2D). Together, these data indicate that despite normal *Mecp2* transcript levels, the *Mecp2* ZFN rat is a viable model to study the consequences of loss of MeCP2 protein.

### Female *Mecp2*^ZFN/+ ^rats model regression and juvenile play deficits

To determine the neurobehavioural consequences of loss of MeCP2 in the rat, we used assays that may be well-suited to testing specifically in the laboratory rat, as well as conventional tests of behaviour in rodents. It is noteworthy that the rat was the model organism primarily used for rodent behavioural studies (45), especially learning and memory test paradigms, prior to the advent of genetic engineering that made manipulations of the mouse genome straightforward. As genetic tools in the mouse evolved, assays used for behaviour in the rat were adapted, developed and improved for comparable studies in the mouse (46,47).

Our studies primarily focused on measurements that were either initiated in juvenile female rats and examined longitudinally through young adulthood, or restricted to juvenile ages. Studying young female *Mecp2*^ZFN/+ ^rats, as opposed to male *Mecp2*^ZFN/y^ rats, is the sex-appropriate disease model for the evaluation of neurobehavioural deficits of clinical relevance to RTT (48). In addition, we selected juvenile ages, in contrast to the adult time points used in the majority of behavioural studies of *Mecp2* mouse models, as the focus of our work was to determine whether female *Mecp2*^ZFN/+ ^rats display behavioural problems during the early stages of life that would better reflect clinical manifestations that occur in young girls with RTT. Two cohorts of female animals were generated to evaluate potential behavioural impairments: one cohort for modelling psychomotor regression, and a separate cohort for investigating the impact of MeCP2 deficiency on multiple behavioural domains.

**Figure 2.**
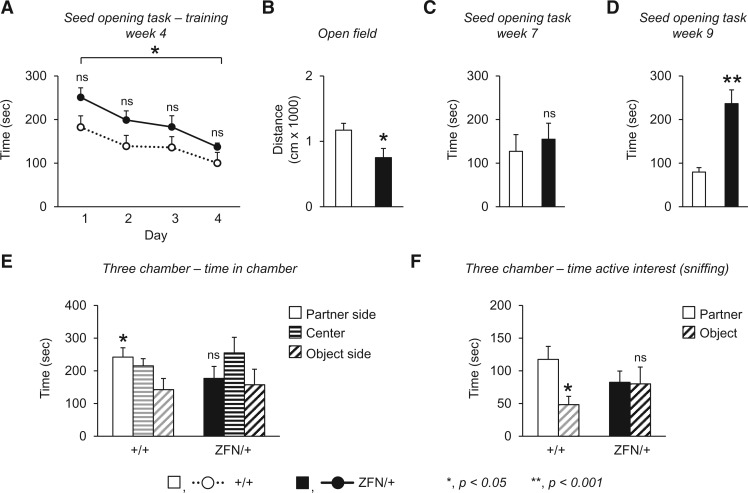
Female *Mecp2*^ZFN/+ ^rats display psychomotor regression and impaired sociability. (**A–D**) Both female *Mecp2*^ZFN/+^rats (ZFN/+) and female wild-type (+/+) littermates were trained in the seed task assay, a test of fine forepaw motor function, at 4 weeks of life, and then evaluated at 7 and 9 weeks of life. Female *Mecp2*^ZFN/+^rats and wild-type littermates performed similarly in their ability to open seeds at 4 weeks of life (A), in spite of reduced general activity in the open field at the same time point as observed in a separate cohort of rats (B). At 7 weeks of life, no difference was observed in the same task (C). At 9 weeks of life, *Mecp2*^ZFN/+^rats appeared to lose the ability of this learned skill, spending significantly more time to open the seeds (D). (**E, F**) In the three chamber test, female wild-type littermates show the expected pattern of normal sociability, spending more time in the chamber containing the novel partner compared with the chamber containing the novel object; in contrast, female *Mecp2*^ZFN/+ ^rats show the conventional definition of impaired sociability (60), spending a comparable time in the chambers containing the novel partner and novel object (E). In addition, female *Mecp2*^ZFN/+^rats do not display a difference in time spent actively investigating, i.e. sniffing, the partner or object (F). *P < 0.05; **P < 0.001; ns, not significant, *n =* 12 per genotype for seed opening task; 9–13 per genotype for three chamber test. A complete statistical summary of behavioural data is provided in Supplementary Material, Table ST12.

Although MeCP2 deficiency in mice results in broad neurobehavioural impairments (29,48–56), it is unclear whether *Mecp2* mouse models display psychomotor regression, a key feature of RTT that is also observed in other IDD/ASD (57). Regression in RTT is clinically defined as the loss of *acquired* skills such as purposeful hand use or language (57). *Mecp2* mouse models show a worsening of specific phenotypes over time, such as generalized exploratory activity in an open arena, or coordination and balance in assays of motor function (49). However, the initial identification and subsequent worsening of a phenotype that model disease *progression* may not specifically reflect psychomotor *regression* in RTT. Therefore, we set out to determine whether *Mecp2* rats displayed regression of an acquired fine motor skill. In a cohort of animals tested longitudinally, naïve young female *Mecp2*^ZFN/+ ^rats and wild-type littermates were trained in a seed opening task at 4 weeks of life, and their ability to open the seeds was evaluated through 9 weeks of life. During the training phase, we found that both groups of rats showed a significant improvement, i.e. reduction in the time spent opening seeds, and similar rate of acquiring the ability to open the seeds across the four days of training (Figure 2A). Furthermore, a significant difference in performance on the first day compared to the fourth day of training was observed independent of genotype (Figure 2A). The ability to acquire and perform in this forepaw skill task was therefore normal for young female *Mecp2*^ZFN/+ ^rats in this cohort of animals tested despite evidence showing reduced generalized exploratory activity in the open field arena (Figure 2B) at the same age of life. To determine whether this acquired skill was maintained over time, rats were tested in a single trial at 7 and at 9 weeks of life. We found that *Mecp2*^ZFN/+ ^rats and wild-type littermates performed equally well at 7 weeks of life (Figure 2C); however, at 9 weeks of life, *Mecp2*^ZFN/+ ^rats showed a loss of this learned skill, spending a significantly longer amount of time to open seeds (Figure 2D). Taken together, these data suggest that psychomotor regression can indeed be modelled in female *Mecp2*^ZFN/+ ^rats.

To test whether *Mecp2* rats can also model additional neurobehavioural features of RTT that have been challenging to study in mice, we evaluated the features of social behaviour in female *Mecp2*^ZFN/+ ^rats and wild-type littermates during the course of studying multiple behavioural domains in a separate cohort of animals not used for the seed opening task study. Although not among the primary diagnostic symptoms of RTT (58), deficits in sociability are prominent, may persist throughout childhood (59), and have been reported in individuals with disease-causing *MECP2* mutations in the absence of a clinical RTT diagnosis (2,17). These clinical findings suggest that impaired social behaviour is an important aspect of MeCP2 disorders. Mice lacking MeCP2, however, demonstrate a reduction in social approach behaviour but do not display the conventional definition of impaired sociability in the three chamber test, an established assay commonly implemented in studies of social behaviour in genetic mouse models (49,60–62). Therefore, we evaluated the performance of juvenile rats in this assay (61) developed for use with mice, and in a social interaction test paradigm that would allow us to take advantage of the rich behavioural repertoire offered by the laboratory rat. In the three chamber test, we found that female wild-type littermate rats exhibit a pattern of normal sociability, spending more time in the chamber side containing the novel conspecific partner compared with the chamber side containing the novel object (Figure 2E); in contrast, female *Mecp2*^ZFN/+ ^rats display the conventional definition of impaired sociability spending a comparable amount of time in the chamber sides containing the partner and object (Figure 2E). Evaluation of the time spent actively interested, i.e. sniffing, the novel partner or object, confirmed the findings from analyzing the chamber time alone (Figure 2F).

**Figure 3.**
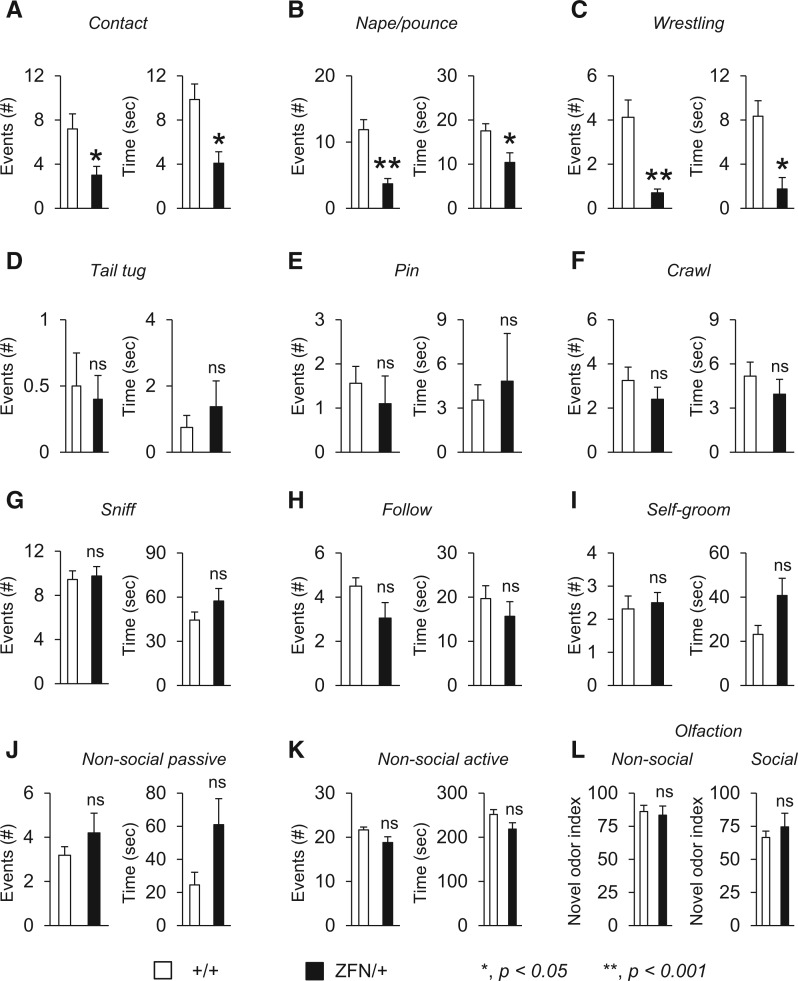
Juvenile female *Mecp2*^ZFN/+ ^rats display selective deficits in aspects of play behaviour. (**A–C**) In a test for direct social interaction, female *Mecp2*^ZFN/+ ^(ZFN/+) rats in comparison with female wild-type littermates (+/+) showed a reduction in the number of events and duration of activity for general paw contact (A), nape/pounce behaviour (B) and wrestling (C) when paired with a conspecific partner rat of the same sex and age. (**D–H**) In contrast, other aspects of play behaviour directed at conspecific partner rats such as tail tugging (D), pinning (E), crawling over-and-under (F), sniffing (G) and following (H) were normal. (**I–K**) As a control, evaluations of self-grooming, non-social passive behaviour and non-social active behaviour such as digging and general exploratory activity demonstrated comparable activity for both female *Mecp2*^ZFN/+ ^and wild-type littermate rats during the test period. (**L**) Furthermore, no differences were observed in social odour discrimination, indicating that the loss of MeCP2 in female rats does not alter gross olfaction. *P < 0.05; **P < 0.001; ns, not significant, *n =* 8–10 per genotype. A complete statistical summary of behavioural data is provided in Supplementary Material, Table ST12.

To further refine the nature of the social behaviour deficit of female *Mecp2*^ZFN/+ ^rats beyond a simple evaluation of indirect social interest, we performed a direct social interaction test that evaluated both general social and juvenile-specific play behaviours (62). Normally, rats in direct contact with each other display social interaction patterns that include age-restricted rough-and-tumble play behaviour (41,43,62,63). We found that juvenile female *Mecp2*^ZFN/+ ^rats engage their conspecific partners less in both generalized contact and specific juvenile play behaviours compared with female wild-type littermate rats (Figure 3A–C). ‘Contact’ behaviour of female *Mecp2*^ZFN/+ ^rats, defined as the test subject’s paw touching the conspecific juvenile partner’s body, was reduced in both the number of events observed as well as the duration of the contact behaviour (Figure 3A). Similarly, female *Mecp2*^ZFN/+ ^rats also displayed a reduction in the number of events and duration of the play behaviours ‘nape/pouncing’ and ‘wrestling’ (Figure 3B,C). In contrast, other play behaviours such as ‘tail tug’, ‘pin’ and ‘crawl’ were normal in comparison to wild-type littermate rats (Figure 3D–F). To ensure the selective deficits in aspects of play behaviour were not due to broad deficits in olfaction, movement, or general activity during the assay, we evaluated sniffing, following/chasing, self-grooming, passive non-social, and active/exploratory non-social behaviours and found that female *Mecp2*^ZFN/+ ^rats did not show differences in these control parameters in comparison to wild-type littermates (Figure 3G–K). In addition, olfaction in both non-social and social odour comparisons revealed normal novel versus familiar odour discrimination indices (Figure 3L), indicating that the impairments in overall sociability and play behaviour were not due to problems in gross olfaction.

### Juvenile female *Mecp2*^ZFN/+ ^rats display additional behavioural abnormalities that are either consistent with or different from previous studies of *Mecp2* mouse models

**Figure 4.**
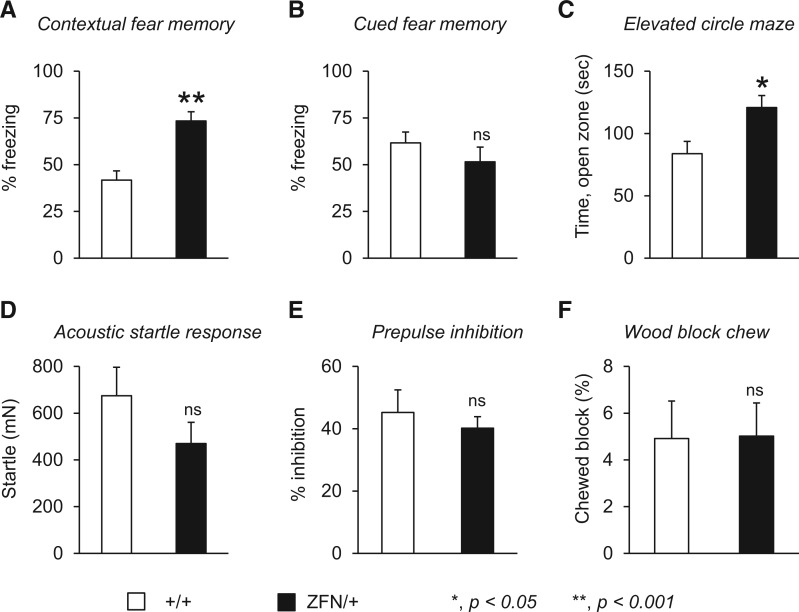
Juvenile female *Mecp2*^ZFN/+ ^rats display alterations in fear memory and anxiety-like behaviour but normal sensorimotor gating and perseverative behaviour. (**A, B**) Juvenile female *Mecp2*^ZFN/+ ^rats (ZFN/+) display increased freezing behaviour compared with female wild-type littermates (+/+) when tested for contextual (A) but not cued fear memory (B). (**C**) Juvenile female *Mecp2*^ZFN/+ ^rats compared with female wild-type littermates spend more time in the open zones of the elevated circle maze. (D, E) Acoustic startle (**D**) and prepulse inhibition of the startle response (**E**) are normal in juvenile female *Mecp2*^ZFN/+ ^rats. (**F**) In a test for perseverative behaviour, juvenile female *Mecp2*^ZFN/+ ^rats and female wild-type littermates do not display differences in chewing a wooden block. *P < 0.05; **P < 0.001; ns, not significant, *n =* 9–13 per genotype. A complete statistical summary of behavioural data is provided in Supplementary Material, Table ST12.

To further identify similarities and/or differences among *Mecp2* rat and mouse models, the female animals evaluated for social behaviour were also tested for impairments in anxiety-like behaviour, sensorimotor gating, learning and memory, and perseverative behaviour. Although fear conditioning studies in *Mecp2* mouse models have demonstrated reduced contextual fear and normal cued fear memory in adult animals (49,51,53,64), we found that juvenile female *Mecp2*^ZFN/+ ^rats displayed enhanced contextual fear memory (Figure 4A), yet normal cued fear memory (Figure 4B). Increased anxiety-like behaviour is one potential confound that may contribute to enhanced freezing behaviour; however, juvenile female *Mecp2*^ZFN/+ ^rats in comparison with wild-type littermate rats displayed reduced anxiety-like behaviour in the elevated circle maze, spending more time in the lit areas of the circle maze compared with wild-type littermates (Figure 4C). Reduced anxiety-like behaviour is consistent with reported studies in *Mecp2* mouse models (49,51) and suggests that the enhanced contextual fear memory observed in young female *Mecp2*^ZFN/+ ^rats is not due to increased anxiety-like behaviour; however, these findings may be potentially confounded by gross motor deficits suggested by the decrease in activity observed in the open field (Figure 2B). Finally, the female juvenile *Mecp2*^ZFN/+ ^rats performed normally in evaluations of sensorimotor gating such as prepulse inhibition of the acoustic startle response (Figure 4D,E), and in the wood block chew assay, a measurement of perseverative behaviour (62) (Figure 4F). A table summarizing the neurobehavioural phenotypes of female *Mecp2*^ZFN/+ ^rats in comparison with the onset and nature of phenotypes in female mice harbouring the *Mecp2-*null mouse alleles (50–53,65) is shown in Table 1.

**Table 1.** Comparison of the juvenile neurobehavioural phenotypes in rodent models of Rett syndrome

	Female juvenile RTT rat	Earliest reported onset in female RTT mice	
	Loss-of-function	Null	
Behavioural domain	*Mecp2* ZFN allele	*Mecp2* Tm1.1Bird (deletion exon3-4) allele	*Mecp2* Tm1.1Jae (deletion exon3) allele
Regression of forepaw use	Normal, 4 & 7 wk; impaired 9 wk	N.E.	N.E.
Sociability	Impaired, ∼ 4 wk	Moderate impairment, 12 wk	N.E.
Anxiety-like behaviour	Reduced, ∼ 3 wk	Reduced, 5 wk	Reduced, 8 wk
Locomotor activity	Reduced, ∼ 4 wk	Reduced, 12 wk	Reduced, 5-6 wk
Sensorimotor gating	Normal, ∼ 5 wk	Altered, 7 wk	N.E.
Contextual fear memory	Enhanced, 5 wk	Impaired, 8 wk	Normal, 6 wk
Cued fear memory	Normal, 5 wk	Normal, 8 wk	Normal, 6 wk
Olfaction	Normal, ∼ 6 wk	Normal, 12 wk	N.E.
Perseverative behaviour	Normal, 6 wk	N.E.	N.E.

Summary of the findings from our studies of juvenile female *Mecp2*^ZFN/+ ^rats compared with published reports on the age of onset and nature of deficits reported in female mice that have either one copy of the *Mecp2*^Tm1.1Bird^ allele (49,50), or one copy of the *Mecp2*^Tm1.1Jae^ allele (51–53). wk, week; N.E., not evaluated or reported.

### Comparative gene expression studies demonstrate the value of evaluating both *Mecp2* rodent models in parallel

**Figure 5.**
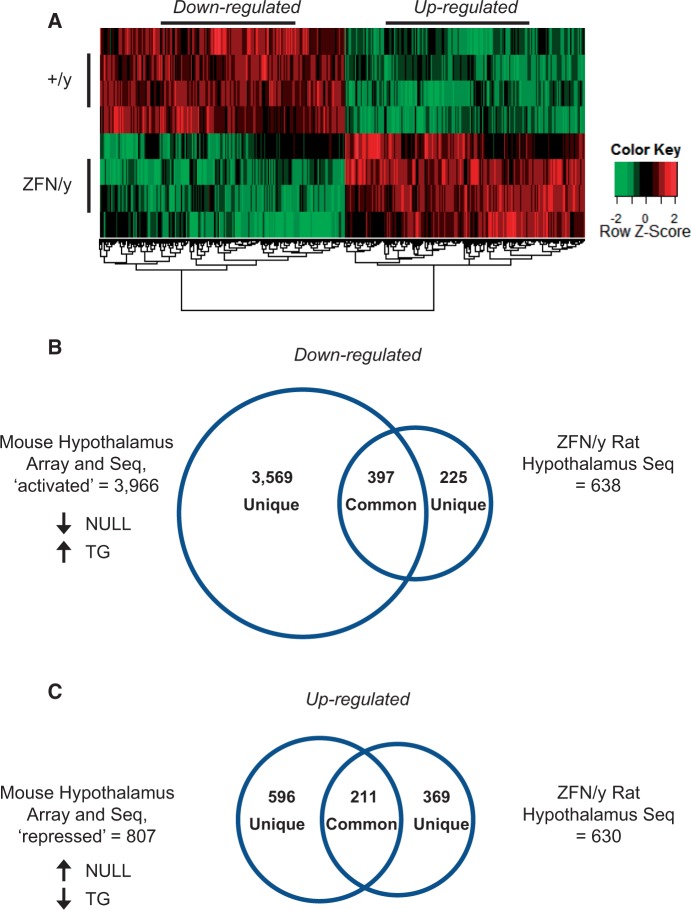
Transcriptional profiling of the hypothalamus using RNA-seq highlights the similarities and differences between transcriptional changes in *Mecp2* rodent models. (**A**) Transcriptional profiling of *Mecp2*^ZFN/y^ (ZFN/y) and wild-type male (+/y) littermate rat hypothalamus reveals an approximate equal distribution of gene expression alterations (638 up-regulated and 630 down-regulated, Bonferroni-adjusted *p* value (*q*) < 0.05). The heat map shown displays baseline expression in +/y animals (top row) relative to altered expression in ZFN/y animals (bottom row) with each row indicating a single animal. (**B, C**) Rat RNA-seq data were compared with existing transcriptional data sets for the hypothalamus of *Mecp2*^Tm1.1Bird/y^ (NULL) and *MECP2-*TG (TG) mice. The overlap in the number of genes altered between *Mecp2* rats and mice is shown, comparing genes down-regulated in rat to genes decreased in NULL/increased in TG, i.e. ‘activated’, defined as decreased in the absence of MeCP2 and increased in the presence of excess MeCP2’ (66,69) (B), and genes up-regulated in rat to genes increased in NULL/decreased in TG, i.e. ‘repressed’, defined as increased in the absence of MeCP2 and decreased in the presence of excess MeCP2 (66, 69) (C). Within these categories, a fraction of genes changed in the ZFN/y rat were either changed in NULL only, changed in TG but not NULL, or changed in other brain regions such as the cerebellum and hypothalamus as previously reported (67, 68), and these changes were either identical or opposite from NULL and TG findings (Table 2). The number of gene expression alterations that were uniquely altered in the MeCP2 rat and mouse models is also shown (B, C). A complete list of gene expression changes in ZFN/y rats identified by RNAseq and compared with existing mouse gene expression datasets is provided in Supplementary Material, Tables ST1-8, gene ontology terms associated with commonly affected gene expression alterations among *Mecp2* rodents and uniquely affected in the *Mecp2* ZFN/y rat are provided in Supplementary Material, Tables ST9, ST10.

To determine whether the *Mecp2* rat model also demonstrates molecular changes that either parallel or differ from those reported in *Mecp2* mice, we compared the transcriptomes of *Mecp2* rats and mice (66–69). For these studies, we reasoned that using male *Mecp2*^ZFN/y^ rats completely lacking MeCP2 would provide a direct comparison with previously published data on *Mecp2*^Tm1.1Bird/y^ mice (66,69), the most commonly used *Mecp2-*null mouse allele (50). We found that in contrast to the thousands of gene expression alterations previously reported in the hypothalamus of *Mecp2*^Tm1.1Bird/y^ mice (NULL) using both array- and sequencing-based technologies, RNA-seq analysis of *Mecp2*^ZFN/y^ rat hypothalamus revealed a total of 1,268 significantly altered gene expression changes (Figure 5A). Similar to previous studies in the mouse, the magnitude of fold changes were not dramatic, with gene expression changes within the range of approximately log_2 _±_ _2 (Figure 5A; Supplementary Material, Tables ST1-8). However, unlike *Mecp2* mouse models that showed a disproportionate number of up-regulated compared with down-regulated gene expression changes in the hypothalamus (66,69), *Mecp2*^ZFN/y^ rats appear to have an approximate equal balance of alterations (638 down-regulated versus 630 up-regulated genes; Figure 5B,C; Table 2).

**Table 2.** Summary of gene expression alterations in *Mecp2* rat hypothalamus and comparison with findings from *Mecp2* mouse hypothalamus

Total expression changes in ZFN/y Rat HYP (#)		SHARED					UNIQUE
		Common to ZFN/y Rat & Mouse NULL/TG HYP				Changed in ZFN/y Rat & Mouse CER or AMY, not HYP	Unique to ZFN/y Rat HYP
			Changed in Rat & TG, not NULL	Opposite from NULL or like TG	Opposite from NULL & TG		
**DOWN**	638	397	29	4	0	16	225
**UP**	630	211	32	22	1	50	369
**Grand total**	1268	608	61	26	1	66	594
	**Percent of total (%)**	47.95	4.81	2.05	0.08	5.21	46.85

Table summarizing the total number of gene expression changes identified by RNAseq in the hypothalamus of *Mecp2*^ZFN/y^ rats (ZFN/y) in comparison with reported findings from either array or RNAseq data in *Mecp2* mice (66–69). Expression changes are categorized according to whether they are ‘shared’ between *Mecp2* rats and mice from our analysis, or are ‘unique’ changes identified only in ZFN/y rats. The genes among the ‘shared’ category indicate the number of genes that are common to the *Mecp2* rat and *Mecp2* mouse (NULL) models, unique to the rat model, changed in the rat and the *MECP2*-TG model (TG), opposite from findings in the NULL or showing an expression difference as observed in the TG, opposite from both NULL or TG, or changed in the rat and in other brain regions of the NULL, but not the hypothalamus.

With respect to the shared gene expression changes between *Mecp2* mouse and rat, approximately one-half of expression alterations (47.95%, or 608 genes) were changed in the same direction as reported in *Mecp2* mouse models. These genes were previously defined as either ‘repressed’ or ‘activated’ according to opposing expression patterns in the hypothalami of NULL mice compared with mice that overexpress MeCP2 (TG) (66,69) (397 down-regulated and 211 up-regulated genes; Figure 5B,C; Supplementary Materials, Tables ST1,ST2). Among this set of commonly altered genes, some alterations in the rat (4.81%, or 61 genes) overlapped with findings in TG mouse hypothalamus alone (66,69), but were not previously reported as altered in NULL mice (29 down-regulated and 32 up-regulated genes; Table 2, and noted in Supplementary Material, Tables ST1, ST2). In addition, only a small fraction of expression changes in the *Mecp2*^ZFN/y^ rat (2.05%, or 26 genes) were directionally opposite of observations in NULL mouse hypothalamus or in the same direction as TG mice (66,69) (4 down-regulated and 22 up-regulated genes; Table 2; Supplementary Material, Table ST3), and only one gene showed an expression difference that was opposite of both *Mecp2* mouse models (Table 2; Supplementary Material, Table ST4). Importantly, however, was the finding that almost half of the gene expression alterations (46.85%, or 594 genes) were uniquely changed in *Mecp2*^ZFN/y^ rat hypothalamus (225 down-regulated and 369 up-regulated genes; Table 2; Figure 5B,C; Supplementary Material, Tables ST5,ST6). Finally, a small number of genes altered in *Mecp2*^ZFN/y^ rat hypothalamus (5.21%, or 66 genes) were not changed in the hypothalamus of either *Mecp2* mouse models, but rather reported to be dysregulated in other brain regions such as the cerebellum (67) and amygdala (68) of *Mecp2* mice (16 down-regulated and 50 up-regulated genes; Table 2; Supplementary Material, Table ST7,ST8). A complete list of gene ontology terms associated with common and unique gene expression alterations is listed in Supplementary Material, Tables ST9,ST10.

To gain further insight into the potential relevance of these gene expression alterations, we evaluated whether genes either commonly changed in both *Mecp2* rat and mouse models (common rat-mouse), uniquely changed in the *Mecp2* rat (unique rat), or uniquely changed in the *Mecp2* mouse models (66,69) (unique mouse) would be predictive of homologous molecular alterations in post-mortem cortical tissue samples from RTT individuals with identified *MECP2* mutations (70). The genes we chose for this analysis were randomly selected from the genes commonly altered in the hypothalamus of *Mecp2* rodents (Supplementary Material, Tables ST1,ST2), uniquely altered in the hypothalamus of *Mecp2*^ZFN/y^ rats (Supplementary Material, Tables ST5,ST6), and uniquely altered in *Mecp2* mouse models (66). In comparison with age- and gender-matched controls, we found that of 82 genes tested, a total of 43 genes (∼52% of the total number of genes tested) were altered or undetectable in the RTT brain by QPCR. The remainder of the genes tested (39 genes, or ∼47% of the total number of genes tested) were not significantly altered in RTT brain (33 genes), or expressed in either RTT or control brains (6 genes).

**Figure 6.**
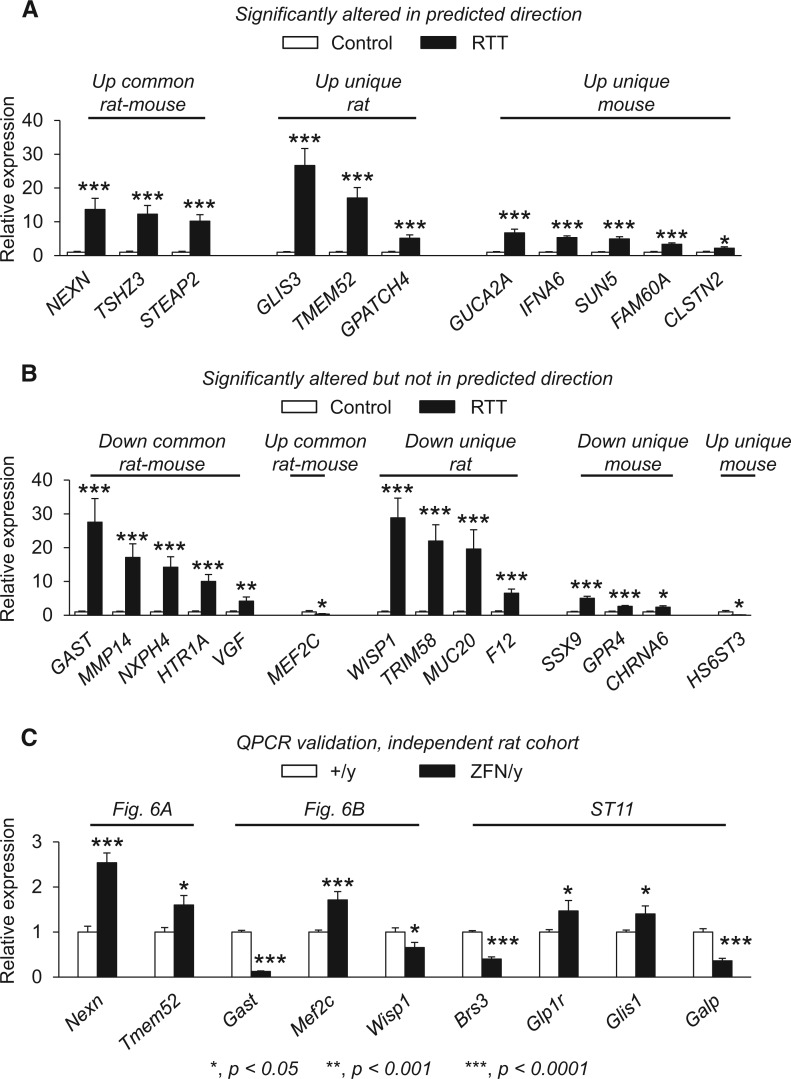
Comparative studies of both *Mecp2* rat and mouse models may strengthen the predictive validity of molecular changes that occur due to the loss of MeCP2 function. (**A, B**) Eight-two genes selected among either the commonly affected alterations between mouse and rat (‘common rat-mouse’), in rat alone (‘unique rat’) or in mouse alone (‘unique mouse)’ were selected for comparative expression studies in human RTT and control brain samples (Brodmann area 9) by QPCR (*n =* 3 RTT; *n =* 4 control). Expression level differences relative to controls samples are shown. Several genes within the up-regulated categories of ‘common rat-mouse’, ‘unique rat’ or ‘unique mouse’ were also altered in RTT brain (A). Additional genes were altered in RTT brain relative to controls; however, these were changed in RTT brain in a direction that was opposite from the predicted *Mecp2* rodent findings (B). Some genes were detected in control brain samples but were not detected in RTT brain. (**C**) As an additional control to ensure that the genes selected for this analysis were indeed altered in *Mecp2* rat hypothalamus, an independent set of animals were used for QPCR validation studies of the top altered genes within each category. All nine genes analyzed were altered as expected in male *Mecp2*^ZFN/y^ rats (ZFN/y) compared with wild-type littermate animals (+/y), confirming the initial RNA-seq findings for this subset of genes. Supplementary Material, Table ST11 shows the expression levels of each gene for both human and rat QPCR data.

To further outline the similarities and differences among the different categories of gene expression alterations between rat, mouse and human, we found that of the 43 expression alterations, 11 genes showed a significant difference between RTT and control brain tissue samples and were altered in a manner predicted by *Mecp2* rodent models. These genes were found to be commonly up-regulated in *Mecp2* rat and mouse hypothalamus (‘up common rat-mouse’; *NEXN, TSHZ3*, and *STEAP2*), uniquely up-regulated in *Mecp2* rat hypothalamus (‘up unique rat’; *GLIS3*, *TMEM52*, and *GPATCH4*), and uniquely up-regulated in *Mecp2* mouse hypothalamus (‘up unique mouse’; *GUCA2A*, *IFNA6*, *SUN5*, *FAM60A*, and *CLSTN2*) (Figure 6A). In contrast, of the 43 gene expression alterations, 14 genes showed a significant difference between RTT and control brain tissue samples but were altered in a manner that was opposite from the *Mecp2* rodent findings. These genes were found to be commonly down-regulated in *Mecp2* rat and mouse hypothalamus (‘down common rat-mouse’; *GAST*, *MMP14*, *NXPH4*, *HTR1A*, and *VGF*), commonly up-regulated in *Mecp2* rat and mouse hypothalamus (‘up common rat-mouse’; *MEF2C*), uniquely down-regulated in *Mecp2* rat hypothalamus (‘down unique rat’; *WISP1*, *TRIM58*, *MUC20*, and *F12*), uniquely down-regulated in *Mecp2* mouse hypothalamus (‘down unique mouse’; *SSX9*, *GPR4*, and *CHRNA6*), and uniquely up-regulated in mouse hypothalamus (*HS6ST3*) (Figure 6B). Finally, 18 of the 43 gene expression alterations had no significantly measureable expression in RTT brain relative to control brain samples, and these genes were distributed among all categories, with the majority (8 of 18 genes) found in the genes classified as commonly down-regulated in *Mecp2* rat and mouse hypothalamus (Supplementary Material, Table ST11). A summary of gene expression data found in RTT brain for each category is provided in Supplementary Material, Table ST11. To ensure that the genes we selected to analyze in human brain tissue samples were indeed significantly altered in male *Mecp2*^ZFN/y^ rats as indicated by initial RNA-seq findings (Supplementary Material, Tables ST1,2,5,6), we tested the top genes that we found altered in RTT brain in an independent set of male *Mecp2*^ZFN/y^ and wild-type littermate rats, choosing a single gene within each category (significantly altered in predicted direction, Figure 6A; significantly altered but not in predicted direction, Figure 6B; and undetectable in RTT brain, Supplementary Material, Table ST11). We found that the nine genes we tested were significantly altered in the hypothalami of male *Mecp2*^ZFN/y^ rats compared with male wild-type littermate animals; thus validating a small subset of the RNA-seq findings from which we based our comparative expression studies (Figure 6C).

## Discussion

With the development of advanced genome editing technologies, such as the ZFN strategy (44) used to generate the *Mecp2* rat model characterized in this study, and even more efficient approaches using CRISPR/Cas gene editing (71–73), generating novel mammalian models of disease beyond the laboratory mouse is feasible. Although some loss-of-function *Mecp2* mouse models of RTT have good construct validity and mimic either the human disease mutation itself or the consequence of the human disease-causing mutations (3,48,74), the findings from our work indicate the value of the *Mecp2* rat model as a powerful tool with the potential to shape the landscape of future studies focused on both mechanisms of RTT pathophysiology as well as on preclinical efforts to ameliorate disease onset and progression.

In this study, our aim was to test the extent to which the loss of MeCP2 in the rat resulted in behavioural deficits present in RTT. However, to confirm that the rat *Mecp2* ZFN allele was appropriate for these studies, we first performed a series of biochemical and molecular evaluations using tissue from both male and female *Mecp2* ZFN rats. Unlike the conventional homologous recombination approach used to generate the most widely studied *Mecp2* germline null mouse model (50), ZFN targeting of the endogenous *Mecp2* locus in the rat did not produce a complete null allele; rather, an allele was created in which normal MeCP2 protein expression was completely abolished yet total abundance of *Mecp2* RNA remained unaltered. Interestingly, although this outcome may not initially appear to be desirable, these results from the rat *Mecp2* ZFN loss-of-function allele are reminiscent of the findings from single-cell, clonally-derived fibroblast strains from RTT girls with disease-causing *MECP2* mutations (75). Indeed, early studies of clonal cell lines from RTT individuals with nonsense mutations showed that *MECP2-*mutant expressing cell lines lacked MeCP2 protein by Western blot analysis using a C-terminal antibody but had normal transcript levels (75). Moreover, the rat *Mecp2* ZFN allele predicted to result in truncated protein did not express any detectable truncation product by either Western blot or antibody staining, similar to observations made with human brain lysates from RTT individuals with truncation mutations (76). In contrast, introducing a late truncating nonsense mutation in the endogenous *Mecp2* locus in the mouse does not entirely parallel these findings in the rat or human. For example, the *Mecp2-*R255X mouse allele (76) resulted in the complete loss of MeCP2 protein and no detectable truncation product, but also caused an approximate 40% reduction in transcript levels. It is noteworthy, however, that the *Mecp2-*T158A mouse allele that disrupts the methyl-CpG binding ability of the protein resulted in a similar scenario as in the rat *Mecp2* ZFN allele; RNA levels were normal despite a loss of protein due to a selective decrease in MeCP2 protein stability (77). Therefore, it is conceivable that humans and rats may share an overlapping post-transcriptional mechanism(s) to regulate the expression of specific mutations in *MECP2*, such as decreased protein stability or perhaps a block in protein translation, that functions differently in mice harboring similar mutation subtypes. Taken together, with respect to our current confirmatory experiments to assess the validity of the rat allele, our data suggest that the rat *Mecp2* ZFN allele has high construct validity that may better reflect the consequences of late-truncating human *MECP2* nonsense mutations.

One primary goal of our neurobehavioural analyses was to identify phenotypic deficits that parallel features found in girls and women with RTT, some of which have not been reported or extensively studied in *Mecp2* mouse models, such as psychomotor regression and impaired sociability. Although there is significant benefit in studying male *Mecp2*^ZFN/y^ rats to understand the molecular mechanisms underlying the complete absence of MeCP2, we reasoned that focusing our behavioural evaluations of young female *Mecp2*^ZFN/+ ^rats would potentially uncover abnormalities in a sex-appropriate animal model that are coincident with the early onset of disease state described in typical RTT.

Psychomotor regression in RTT, defined as the loss of acquired skills such as purposeful hand use, has yet to be well-modelled in *Mecp2* mice. Although longitudinal studies have shown the manifestation and subsequent worsening of phenotypes such as reduced motor function in *Mecp2* mice (49), and one study of symptomatic 10–12 month old *Mecp2-*308 females showed deficits in forepaw dexterity at a single time point late in life (78), the seed opening task (79) adapted for this study allowed us to evaluate the extent to which trained, young female *Mecp2*^ZFN/+ ^rats lose their ability to perform over a period of time through early adulthood. Remarkably, we found that *Mecp2*^ZFN/+ ^rats performed similarly to wild-type littermates on this task during the training phase at 4 weeks of life, and during the single test trial at 7 weeks of life, suggesting that not only did they acquire and learn the skill over time, but also maintained this learned forepaw ability. However, at 9 weeks of age, female *Mecp2*^ZFN/+ ^rats lost their ability to perform well on this task, providing evidence of a phenotype reminiscent of psychomotor regression of an acquired forepaw skill. Here, it is important to note the distinction between disease ‘progression’ versus ‘regression’. Although we cannot formally rule out the possibility of an element of disease progression in the longitudinal seed opening test paradigm at this time, our findings appear to model aspects of regression-like behaviour (learning, acquisition, maintenance and eventual loss of the skill). These data suggest female *Mecp2*^ZFN/+ ^rats demonstrate high face validity for psychomotor regression, an aspect of RTT that not yet been fully appreciated in *Mecp2* mouse models to the extent demonstrated in the current study. Similar longitudinal studies using a forepaw training paradigm in the various *Mecp2* mouse alleles may provide additional insight into the relationship between *Mecp2* mutation subtype, and disease onset and severity in the context of psychomotor regression. However, it remains to be determined whether *Mecp2* mice will perform sufficiently well in the seed opening task. Furthermore, because regression in RTT may also coincide with the loss of speech, it may be informative to determine whether the type and nature of ultrasonic vocalizations (USV) emitted by female *Mecp2*^ZFN/+ ^rats, or other aspects of communication, change over time. Although we did not find differences in the total number of USV emitted by juvenile female *Mecp2*^ZFN/+ ^rats during direct social interaction tests (data not shown), a recent study of symptomatic 5-11 month old female *Mecp2*^ZFN/+ ^rats found alterations in speech processing (80), suggesting that communication deficits, in a broader context, may be affected in this model.

Although there is debate about social behaviour abnormalities in RTT (59), impaired social behaviour in RTT that is consistent with the social deficits of clinically defined autism appears to coincide with stages of early disease progression (81). In our social behaviour studies, we found that young female *Mecp2*^ZFN/+ ^rats displayed both the conventional definition of impaired sociability in an indirect assay, the three chamber test, and selective deficits in aspects of play behaviour. Unlike *Mecp2* mice that have only shown a relative reduction in social interest in the three chamber test, female *Mecp2*^ZFN/+ ^rats indeed show a phenotype that is consistent with findings from other ASD mouse models with impaired sociability. Furthermore, by refining our social behaviour studies to focus on play behaviour (41), an age- and species-specific complex set of behaviours that is only present at a rudimentary level in the mouse (43) and is genuinely present in rats, we found that female *Mecp2*^ZFN/+ ^rats display juvenile play deficits similar to those observed in other ASD rats such as the *Fmr1* (62) and *Nlgn3* (62) ZFN models. Despite the potential confound of reduced motor/exploratory activity as may potentially be suggested by the open field data (Figure 2B), measures quantified during the direct social interaction test, such as evaluating the number and duration of ‘following’, ‘self-grooming’ and ‘non-social active’ exploratory behaviours, provided an additional layer of control (Figure 3H,I,K). Normal levels of activity with respect to these control measures in both female *Mecp2*^ZFN/+ ^and wild-type littermate rats while the animals were actively engaged in this task, as well as normal olfaction with both non-social and social odour cues (Figure 3L), strengthens the interpretation that female *Mecp2*^ZFN/+ ^rats have impairments in selective aspects of play behaviour. Although the biological pathways regulating play behaviour in these rat models are unclear at this time, these data provide an inroad into understanding the neuroanatomical determinants of sociability in genetically tractable models that have a complex repertoire of behavioural phenotypes. These social behaviour studies together suggest social deficits that manifest early in RTT, especially juvenile play behaviour which cannot be studied well in mice, may be more appropriately modelled in young female rats lacking MeCP2.

In addition to identifying novel phenotypes in the *Mecp2* ZFN rat model, we also addressed whether behavioural defects previously reported in *Mecp2* mice were also present in the rat. Evaluations of fear conditioning, anxiety-like behaviour, sensorimotor gating and perseverative behaviour revealed changes in juvenile female *Mecp2*^ZFN/+ ^rats that were either consistent with or different from previous studies of *Mecp2* mice. For example, decreased exploratory activity in the open field arena and reduced anxiety-like behaviour in the elevated circle maze were two phenotypes consistent with known *Mecp2* mouse behavioural data; however, our findings suggest that these phenotypes emerge earlier in life in female *Mecp2*^ZFN/+ ^rats compared with *Mecp2* mice. In contrast, the juvenile female *Mecp2*^ZFN/+ ^rats were normal in tests for perseverative behaviour and sensorimotor gating, both of which have been reported to be altered in *Mecp2* mice (49,82). This is not entirely unexpected, given the difference in the ages of the animals that were tested in this study in comparison with previous work. Interestingly, juvenile female *Mecp2*^ZFN/+ ^rats also displayed an increase in freezing in fear conditioning that was suggestive of enhanced contextual fear memory. Because we identified reduced anxiety-like behaviour in female *Mecp2*^ZFN/+ ^rats, increased anxiety is unlikely a contributor to the increased freezing behaviour after conventional Pavlovian fear conditioning; in contrast, reduced activity observed in the open field may contribute to this phenotype. Reduced contextual fear memory is a hallmark of *Mecp2* mouse models; therefore, it remains to be determined whether identical learning and memory phenotypes can be studied in *Mecp2* rats. It would be informative to study how our current behavioural findings compare with phenotypes present in adult female *Mecp2*^ZFN/+ ^rats, as well as how *Mecp2* rats and mice of the same age would perform using the identical fear conditioning training protocol. These future studies using female *Mecp2*^ZFN/+ ^rats may also benefit from evaluating performance in other learning and memory tasks that do not rely on an aversive training stimulus.

Finally, our comparative studies of gene expression alterations in *Mecp2* rats and mice and their predictive validity in RTT post-mortem brain tissue revealed several key similarities and differences among the two rodent models. Previous profiling strategies of *Mecp2* mice employed microarray and more recently, sequencing-based technologies (66,69). Because strong evidence provided by multiple studies including those led by the Sequencing Quality Control/Microarray Quality Control consortium suggests that there is good concordance across gene expression platforms (83–87), such as array versus sequencing-based approaches, we chose to compare our data to the complete list of genes altered in *Mecp2* mice from both array and RNA-seq studies. The ‘union’ of genes expression data, i.e. the total aggregate of all gene expression changes identified in both hypothalamus array (66) and sequencing-based studies (69), from *Mecp2* mice allowed us to identify all possible overlapping hypothalamic gene expression changes shared with the *Mecp2*^ZFN/y^ rats and provide an unbiased reference point that did not rely solely on findings from a single profiling approach. This strategy also ensured that the remaining genes that are not commonly altered within the hypothalamus of *Mecp2* rodents are indeed uniquely changed in male *Mecp2*^ZFN/y^ rat. Interestingly, one notable similarity is that the fold expression changes in both *Mecp2* rodents were of the same magnitude (±2 fold, log_2_ scale). Although this magnitude of fold change has also been reported in other brain regions of *Mecp2* mice such as the cerebellum (67) and amygdala (68), as well as the striatum (88), it would be useful to determine whether additional brain regions of the *Mecp2* rat are similarly affected. Moreover, although approximately half of the genes identified as altered in male *Mecp2*^ZFN/y^ rats were commonly altered in *Mecp2* mice, the majority of these commonly affected genes were directionally concordant between rat and mouse in terms of up- or down-regulation. Together, these data may indicate that the loss of MeCP2 has strikingly similar effects on gene expression for a subset of genes among divergent rodent species. With respect to differences between gene expression alterations among *Mecp2* rodent models, approximately half of the expression changes we identified are unique to the rat; these findings may have important implications in the manifestation of behavioural phenotypes that are present in the *Mecp2* rat model.

Lastly, working with the limitations of available human post-mortem tissue, our approach to use *Mecp2* rodent data to predict changes in RTT was revealing. By testing shared or unique *Mecp2* rodent gene expression changes in RTT and control brains, we found that among the genes altered in RTT brain, the only genes that were predictive of changes in RTT brain were up-regulated in either both *Mecp2* rodent models or each rodent model alone. Although additional comparative profiling would be required, these initial data may possibly support the model of MeCP2’s historical role as a transcriptional repressor of genes (89) with specific relevance to human disease pathogenesis. In contrast, the genes that were altered in RTT brain but not in the same direction as predicted from either *Mecp2* model appeared to be genes mostly representing the subsets of down-regulated genes. Interestingly, these alterations had the highest relative fold changes in RTT brain. Despite the limited number of genes analyzed by conventional QPCR and the differences in brain region analyzed (rodent hypothalamus compared with human Brodmann area 9 containing the frontal cortex), it is noteworthy we identified concordant gene expression changes in *Mecp2* rodent models and RTT brain samples. Our additional validation QPCR studies in an independent set of male *Mecp2*^ZFN/y^ rats and male wild-type littermate animals confirmed the top altered genes from each category, including those that were not detectable in RTT brain, suggesting that these changes, at least for this small subset of genes, are genuine alterations in the *Mecp2* rat model. Taken together, these comparative data strongly suggest that the combination of analyzing both loss-of-function *Mecp2* rodent models may provide a significant advantage for identifying transcriptional changes and likely other biochemical and molecular changes that occur in the human RTT brain that may not have been predicted in analyses of either rodent model alone. In the context of recent findings suggesting that MeCP2 may regulate the expression of long genes in a cell-type and brain-region specific manner (90,91), and preferentially bind to DNA at methylated cytosine at CG sites (92) yet also bind to methylated cytosine at non-CG sites (69) as well as nucleosomal sequences with high GC content alone (93), it would be highly informative to pursue similar studies using the *Mecp2* rat model to further clarify the role of MeCP2 in the epigenetic regulation of gene expression and its impact on disease features of RTT and related disorders.

In conclusion, our work demonstrates the utility of the rat *Mecp2* ZFN allele for identifying molecular alterations and neurobehavioural outcome measures that may be useful for future preclinical studies. In addition, our comparative gene expression studies demonstrate the strength of combining *Mecp2* rodent data sets to potentially improve the predictive validity of alterations that occur in RTT, some of which may have greater disease-relevance. Given the perceived shortcomings of using rodent models for preclinical validation studies of therapeutic compounds for use in humans (36,94), and the urgent need for outcome measures to expedite the discovery of successful therapies (48), this work underscores the significant benefits of investigating additional mammalian rodent models of RTT, and provides a framework for studying features provided by the *Mecp2* rat model that will complement ongoing mouse studies.

## Materials and Methods

### Animal husbandry

All research and animal care procedures were approved by the Baylor College of Medicine Institutional Animal Care and Use Committee, and all studies detailing our findings were conducted at Baylor College of Medicine. Rats were maintained on a 12 h light:12 h dark cycle with standard rat chow (Pico Lab Rodent Diet, #5053, Purina USA) and water ad libitum. Animals were generated by mating female Sprague-Dawley rats lacking one copy of functional MeCP2 (*Mecp2*^ZFN/+^) with wild-type male Sprague-Dawley rats (SAGE Labs/Horizon, USA) to obtain male rats completely lacking MeCP2 (*Mecp2*^ZFN/y^), female *Mecp2*^ZFN/+ ^rats and wild-type littermate male and female rats. Rats were housed three animals per cage, and randomized and blinded for subsequent experiments described below. Genotyping was performed in accordance with the manufacturer’s protocol (SAGE Labs/Horizon, USA) using the following primers to identify animals that harbour the ZFN allele: forward, 5’-GCAGC ATCA GAAGGTGTTCA-3’; reverse, 5’-GACCTCAATGCTGACGGTTT-3’. All wild-type animals used in the study were age- and sex-matched littermates of the generated male and female *Mecp2* rats; conspecific female wild-type partner rats (purchased from SAGE Labs/Horizon, USA) used in the direct social interaction study as described below, were not littermate animals.

### Western blot

Fresh brains were dissected from rat cortex samples (*n =* 4 biological replicates of each genotype, *Mecp2*^ZFN/y^ and *Mecp2*^ZFN/+^, and respective wild-type littermates, 6 weeks of life). The brains were homogenized in 100 mM Tris, pH 8.0, and 2% SDS with protease inhibitor cocktail (Sigma-Aldrich, USA) in a glass dounce homogenizer with the B handle and rocked at room temperature for at least 1 h to ensure complete cell lysis. Soluble proteins were separated from cell debris by centrifugation at 20,000*g* for 15 min, and the supernatant was quantified using a colorimetric assay (Pierce BCA Protein Assay Kit; ThermoFisher Scientific, USA). Forty µg of protein per sample were loaded onto a PAGE gel and transferred onto a nitrocellulose membrane. The membrane was blocked in Tris-buffered saline, pH 7.6, and 0.1% Tween 20 with 5% milk. For immunodetection of MeCP2, an antibody raised in rabbit (95), diluted 1:2000 was used; detection of the loading control GAPDH was performed with an antibody raised in mouse (sc-32233; Santa Cruz Biotechnology, Inc.; USA) diluted 1:20,000. Membranes were incubated for 1 h with Cy5-conjugated secondary antibodies against mouse and rabbit diluted 1:4000 (GE Healthcare Life Sciences), imaged using the Cy5 channel (ImageQuant LAS 4000, GE Healthcare Life Sciences) and quantified by densitometry with ImageJ software as previously described (76).

### Quantitative real time reverse transcriptase PCR

For QPCR analysis of *Mecp2e1* and *Mecp2e2* in rat, RNA was extracted from rat cortex samples (*n =* 4 biological replicates of each genotype, *Mecp2*^ZFN/y^ and *Mecp2*^ZFN/+^, and respective wild-type littermates, 6 weeks of life) using TRizol reagent following the manufacturer’s instructions (ThermoFisher Scientific, USA), and 3 µg RNA was used for cDNA synthesis (iScript cDNA synthesis kit, Bio-rad, USA). QPCR using SsoAdvanced Universal SYBR Green Supermix (Bio-rad, USA) was performed using the standard curve method with rat *Mecp2e1-* and *Mecp2e2-*specific (96) and rat *Gapdh* primers, and expression level of each *Mecp2* isoform was quantified relative to wild-type littermate control animals as previously described (68). For QPCR analysis using human RTT and control brain tissue samples (*n =* 3 RTT, *n =* 4 control; Brodmann area 9 used in previous reports (70,97)), similar methods were used for RNA extraction and 1 µg of RNA was used for cDNA synthesis. QPCR using SsoAdvanced Universal SYBR Green Supermix (Bio-rad, USA) was performed using 96-well plates seeded with a commercial primer assays for 82 genes of interest and controls (PrimePCR, Bio-rad, USA), and relative expression levels comparing RTT to control brain samples were analyzed using commercially available software according to the manufacturer’s instructions (Bio-rad CFX Manager, Bio-rad, USA). Validation QPCR studies were also performed similarly using commercial primer assays for the 9 genes of interest and control *Gapdh* (PrimePCR, Bio-rad, USA); the top 9 genes among the different categories as described in the main text were selected on the basis of availability of assays, *Glis3* was not available and therefore we analyzed *Tmem52* as the next representative gene of the category ‘up unique rat’. Validation studies were conducted using hypothalamus obtained from an independent set of male *Mecp2*^ZFN/y^ (*n =* 5) and wild-type littermate rats (*n =* 4) at approximately 6 weeks of life.

### Immunofluorescence staining

Fresh frozen tissue was embedded in optimal cutting temperature medium and cut into 25 μm sections onto slides. Tissue was fixed on the slide with 4% paraformaldehyde in phosphate buffered saline (PBS) for 30 min, washed with PBS, permeabilized with 0.3% Triton-X 100 (PBST) for 15 min, and blocked for 2 h in 10% normal goat serum with 0.3% Triton-X 100 in phosphate buffered saline (blocking buffer). Samples were then incubated with primary antibody in blocking buffer overnight, washed with PBST, incubated in secondary antibody in blocking buffer for 4 h, incubated with 0.2 μg/ml DAPI in PBST, and mounted with coverslips using Prolong Gold Antifade Mounting Medium (ThermoFisher Scientific, USA), with wash steps in PBST before each incubation procedure, and final mount step. Primary antibodies used included Chicken anti-MeCP2 (ABE171 diluted 1:500; Millipore, USA), and Rabbit anti-NeuN (ABN78 diluted 1:500; Millipore, USA). Secondary antibodies used included Alexa Fluor conjugated Goat anti-Chicken and Goat anti-Rabbit antibodies (1:500; Jackson ImmunoResearch, USA). Image acquisition was performed using a Zeiss 710 confocal microscope, and prepared with ImageJ as previously described (98). Single animals of each genotype (*Mecp2*^ZFN/y^ and *Mecp2*^ZFN/+^, and respective wild-type littermates, 6 weeks of life) were used to generate the representative images shown in Figure 1; for further quantification of MeCP2 signal intensity and percentage, additional animals were analyzed as described in Supplementary Materials, Text and Figure S3.

### Rat neurobehavioural assays

Previous work revealed that young Sprague-Dawley rats exhibited significantly decreased activity under bright light conditions (62), which could interfere with the outcome of behavioural assays. Therefore, all tests were carried out under dim-lighting conditions between 10 and 25 lux as previously described (62). Starting at approximately 24 days of life, rats were subjected to a behavioural battery consisting of the following assays described below. For each assay, rats were habituated to the test room for at least 30 min (60 dB and lux level as indicated below for each test). Behavioural analysis was performed only with female rats in this study. Two separate cohorts of female rats were generated as described above; one cohort consisting of female *Mecp2*^ZFN/+ ^(*n =* 12) and wild-type littermate rats (*n =* 12) for evaluation in the seed opening task, and one cohort consisting of female *Mecp2*^ZFN/+ ^(*n =* 9) and wild-type littermate rats (*n =* 13) for evaluation in elevated circle maze, three chamber test for sociability, direct social interaction, open field activity, acoustic startle response and prepulse inhibition of the startle response, fear conditioning, olfaction and wood block chew test. The cohort tested for multiple behavioural domains was tested first over a period of 6 months total; approximately 1–1.5 year later, the separate cohort tested longitudinally for the seed opening task was tested for a period spanning approximately 6–7 months. For both cohorts, animals were tested in batches as they became available, with 5–6 animals maximum per group. All animals were tested in a randomized fashion; each experiment was performed by the same individual blinded to genotype, including video scoring. In the cohort of animals tested for direct social interaction, all animals were tested; however, four video files were not recovered and only female *Mecp2*^ZFN/+ ^(*n =* 10) and wild-type littermate rats (*n =* 8) were included in the analysis. All other tests that were performed included all animals in the analysis.

### Seed opening task

Psychomotor function related to forepaw usage was evaluated by training rats in their ability to open sunflower seeds as previously described (79,99). At 4 weeks of life, in the evening prior to the first day before food deprivation, sunflower seeds were placed in the home cage of rats that were to be tested to expose them to seeds prior to any testing. Animals were food-deprived overnight prior to each training session (approximately 20 h). On training days, animals were subjected to a training period consisting of a 5 min test session performed once per day for four consecutive days. Test subjects were placed in the centre of the cylinder, and were allowed to manipulate and open five sunflower seeds randomly placed in the cylinder. Test subjects were trained in a transparent plexiglass cylinder (22.8 cm, height; 16.5 cm, diameter). A mirror was placed behind the cylinder to facilitate the viewing and scoring of behaviour. At 7 and 9 weeks of life, tests were performed, one single trial per time point, similarly as described above, including overnight food-deprivation; however, if test subjects spent greater than 5 min to manipulate and open seeds during any given test trial, then 300 s was recorded.

### Elevated circle maze

At 24 days of life, anxiety-like behaviour was tested on an elevated, circular platform (100) with 2 closed and 2 open regions (39 cm, height; 60 cm, diameter; 6.3 cm, platform width; 15 cm, closed zone wall height; 46 cm, closed zone wall length). Test subjects were placed in one of the open regions and allowed to freely explore the platform. The time spent in the open and closed regions, and behaviours including grooming and rearing were recorded for 10 min in dim lighting conditions (10 lux). Behavioural scoring was performed in real-time by an experimenter blinded to genotype using a hand-held computer (Psion Observer 3, Noldus, USA).

### Three chamber test for sociability

At 25 days of life, rats were tested in an apparatus composed of three chambers (two side chamber and a centre chamber with doorways to both side chambers, all of equal dimensions (42.5 cm, length; 17.5 cm, width; 23 cm height). As a modification of the original apparatus (61) to accommodate the juvenile rats, inverted cups were replaced by creating two small chambers within the side chambers as previously described (62). The two small chambers (10 cm, length; 17.5 cm width; 23 cm height) were created with perforated plexiglass partitions that allowed rats to see, hear and smell across the walls. The evaluation of sociability consisted of two 10 min test sessions, a habitation phase and a test phase performed in dim lighting conditions (15 lux). During the habituation phase, each test subject was placed in the centre chamber and allowed to freely explore the apparatus for 10 min. Test subjects were then allowed to return to the centre chamber and the entrances to the side chambers were blocked with plexiglass walls. Either a conspecific novel wild-type partner rat of the same genetic background, age and sex (purchased from SAGE Labs/Horizon, USA), or a novel object (a light grey LEGO block, 6 cm, length; 6 cm, width; 4 cm, height) was immediately placed behind the plexiglass partitions of the side chambers. The walls obstructing the entrances to the chambers were lifted and the test phase was initiated in which test subjects were allowed to freely investigate all three chambers. The entire test was video recorded, and time spent in each chamber (partner side, centre and object side), as well as time spent actively investigating the wild-type partner or novel object (sniffing at the partition) were scored by an experimenter blinded to genotype using a hand-held computer as previously described Psion Observer 3, Noldus, USA).(62).

### Direct social interaction

At 27 days of life, test subjects were single-housed in a clean standard rat cage (40.6 cm, length; 20.3 cm, width; 19 cm, height) with bedding for 3 h prior to testing in dim lighting conditions (10 lux). A novel wild-type partner rat of the same genetic background, age and sex (purchased from SAGE Labs/Horizon, USA) was then placed in the clean cage and animals were allowed to freely engage each other for 10 min. Behaviours were video recorded and the number of events and time spent engaged in specific behaviours were quantified by an experimenter blinded to genotype using a hand-held computer (Psion Observer 3, Noldus, USA). The evaluation of distinct behavioural categories was defined according to previous studies of juvenile play behaviour in rats (41,62,63). *Contact behaviour* was defined as general contact of the test subject (either female *Mecp2*^ZFN/+ ^rat or wild-type littermate) with the partner rat, such as test subject paw placed on the partner body or face with no additional behavioural activity such as sniffing. *Nape/pounce* behaviour was defined as the test subject pouncing on the partner rat with activity from the test subject’s face and/or paws directed towards the nape of the partner rat. *Wrestling* behaviour was defined as events in which both animals push, paw and/or grab each other. *Tail tug* behaviour was defined as the test subject pulling and/or tugging the partner rat’s tail. *Pin* behaviour was defined as the test subject standing directly over the partner rat with its dorsal surface touching the test chamber floor, rendering the partner rat immobile. *Crawl* behaviour was defined as the test subject crawling over and under the partner rat. *Sniff* behaviour was defined as the test subject sniffing the partner rat during stationary encounters with no additional movement. *Follow* behaviour was defined as the test subject actively sniffing and following/chasing the partner rat. Additional measurements of behavioural activity were evaluated as control parameters. *Self-grooming* behaviour was defined as the test subject self-grooming areas of its body and tail with either one or both paws. *Non-social passive* behaviour was defined as the test subject neither interacting with the partner rat nor participating in active exploratory behaviours. *Non-social active* behaviour was defined as the test subject generally exploring their test environment without interacting with the partner rat.

### Open field activity

At 29 days of life, activity was assessed by measuring the total distance travelled in a plexiglass arena (40 cm, length; 40 cm, width; 30 cm height) equipped with photobeams (Accuscan Instruments, Columbus, OH, USA) for 15 min as previously described (62). Testing was performed under dim lighting conditions (15 lux).

### Prepulse Inhibition of the acoustic startle response

At 32 days of life, acoustic startle response (ASR) and prepulse inhibition of the ASR were assessed using the SR-Lab System (San Diego Instruments, San Diego, CA, USA). The apparatus was composed of a sound attenuating chamber, which contained a cylindrical tube where the test subject was placed during testing. Each animal was acclimated to a background white noise of 70 dB for about 5 min prior to the test session. In order to habituate the animals, 10 startle only stimuli were presented at the beginning of each test session as previously described (62). Each test session consisted of 48 trials (six blocks of eight trial types each presented in a pseudo-random order). Each block had a ‘no stimulus’ trial used to measure baseline movement where no sound was presented, a ‘startle only’ trial composed of a 40 ms, 120d B sound burst, ‘prepulse only’ trials (20 ms prepulses of 74, 78 or 82 dB sound) and ‘prepulse inhibition (PPI)’ trials composed of the presentation of one of the three prepulse sounds 100ms prior to the startle stimulus of 120 dB sound. The inter-trial interval ranged from 10–20 s, and the startle response was recorded every 1 ms for 65 ms following the onset of the startle stimulus. Percent PPI of the ASR was calculated as follows: 100 – [(response to acoustic prepulse plus startle stimulus trial/the startle response alone trial) × 100].

### Fear conditioning

At 34 and 35 days of life, animals were subjected to classical fear conditioning as previously described (62). Test subjects were conditioned in a chamber (29.2 cm, length; 24.5 cm, width; 21.5 cm, height) composed of a rectangular box with a metal grid floor (Med Associates Inc., St. Albans, VT, USA). Test subjects were placed in the chamber for 2 min, then exposed to one conditioned stimulus-unconditioned stimulus (CS-US) pairing of a 30 s, 80dB sound (CS) followed by a 2 s, 1 mA foot shock (US). Twenty-four hours later, test subjects were evaluated for both context and cued memory. For contextual fear memory, test subjects were placed in the test chamber for 5 min and the time spent freezing was measured. One hour later, cued fear memory was evaluated. Test subjects were placed in a novel test chamber modified with one white opaque plexiglass sheet placed on the floor of the chamber, and a second white opaque plexiglass sheet placed diagonally to divide the chamber into two compartments. Test subjects were placed in one half of the divided chamber, and a small dish containing vanilla extract was placed in the second half of the chamber. The time spent freezing was measured for 3 min without auditory stimulus, followed by 3 min of continuous auditory stimulus. Time spent freezing for both context and cued fear memory was analyzed using automatic detection software (FreezeFrame version 2.0, Actimetrics, Wilmette, IL, USA) and expressed as a percentage.

### Olfaction test

At 40 and 41 days of life, test subjects were evaluated for gross olfaction using non-social and social odours. Testing was performed in an open field chamber as described above in dim lighting conditions (15 lux). The floor of the chamber contained 16 holes that were baited with various odours placed in small plastic dishes (2 cm, length; 2 cm, diameter). The holes allowed test subjects to smell but not touch odour cues. On the first day of testing, test subjects were placed in the chamber containing the first non-social odour cue (cilantro paste) in three of the 16 holes. The time spent sniffing the holes containing the non-social odour cue was recorded for 3 min. One of the three holes containing the non-social odour cue was then replaced with a second, novel non-social odour cue (rosemary), and the time spent sniffing the holes was measured for 3 min. The following day, the test was repeated using social odour cues. Test subjects were placed in the chamber containing the first social odour cue (small, plastic dish with bedding material from the test subject’s home cage) in three of the 16 holes, and the time spent sniffing the holes was measured for 3 min. One of the three holes containing the first social odour cue was then replaced with a second, novel social odour cue (small plastic dish with bedding material from a cage of unfamiliar rats of the same sex), and the time spent sniffing the holes was measured for 3 min. The duration of sniffing at all holes was recorded by an experimenter blinded to genotype using a hand-held computer (Psion Observer 3, Noldus, USA). For both non-social and social tests, the time spent sniffing the novel odour was compared with the familiar odour and expressed as a novel odour index (time spent sniffing novel odour/time spent sniffing familiar odour × 100).

### Wood block chew test

At 42 days of life, test subjects were single-housed overnight from 16:00 – 09:00 in a clean standard rat cage with a non-toxic, plain wood block (Aspen wood; 0.4 cm, length; 0.2 cm, width; 0.2 cm, height). Wood block mass was recorded before and after the overnight exposure as a measurement of perseverative-like behaviour as previously described (62).

### Statistical analyses of behavioural, western and QPCR data

Statistical analyses of data were performed using a commercially available statistical software package (SPSS, version 23), with the exception of seed opening task data. Data from the elevated zero maze, open field, acoustic startle, prepulse inhibition, wood block chew, fear conditioning, olfaction, direct social interaction, and Western blot and QPCR studies were analyzed using a one way analysis of variance (ANOVA) with genotype as a factor. For testing sociability in the three chamber test, data related to time spent actively investigating the partner or object were analyzed using a one way ANOVA with genotype as a factor. Data related to time spent in each chamber (partner chamber, centre, and object chamber) were analyzed using a one way ANOVA with genotype as a factor; the time spent in the centre compartment is shown for illustrative purposes in the graph but not included in the statistical analysis as previously described (60). Seed opening task was analyzed using standard statistical procedures in GraphPad Prism for linear regression analysis, two-way ANOVA and multiple comparison *post-hoc* testing with Sidak’s procedure.

### RNA sequencing and analysis

RNA extraction (*n =* 4 biological replicates of each genotype, male *Mecp2*^ZFN/y^ and male wild-type littermate hypothalamus, 6 weeks of life) was performed as described above and 10 µg prepared for pair-end RNA-Sequencing using Illumina HiSeq 2500. All sequencing was performed by the Genomic and RNA Profiling Core at Baylor College of Medicine. For each sample, about 70 to 90 million of 100bp pair-end reads were generated. The raw reads were aligned to the *Rattus norvegicus* genome (Ensembl *RGSC3.4*) using TopHat v1.4.1 (101) with default parameters (*-r 400 –p 8*). The first 10 base-pairs of each read at the 5’-end were trimmed prior to mapping to a reference genome. The mappability for each sample was all above 85%. To prepare the aligned sequence reads into an expression level for differential gene analysis, we employed the free Python program HTSeq (102) for this purpose. The *htseq-count* function of HTSeq allowed us to accumulate the number of aligned reads that falls under the exons of the gene (union of all the exons of the gene). These read counts obtained are analogous to the expression level of the gene.

Using the obtained raw counts, differential gene analysis was carried out using the DESeq package in the R environment. DESeq includes functions for us to test for gene expression changes between samples in different conditions by the use of the negative binomial distribution and a shrinkage estimator for the distribution’s variance (103). The *nbinomTest* function of the package was used to test if a gene is significantly differentially expressed. Benjamini-Hochberg False discovery rate (FDR) adjusted p-values are reported. Heatmap of the differentially expressed genes (FDR < 0.05, at least 1.5 fold-changes) were plotted. The plotted expression (z-scores) for each gene was the expression values normalized at the gene level to have a mean of zero and standard deviation of one. The heatmap was drawn based on the normalized expression data clustered by both gene and samples based on Euclidean distance and complete linkage. Gene ontology analysis was carried out with DAVID (http://david.abcc.ncifcrf.gov/) using the mouse gene symbols. The lists of genes were queried in DAVID in order to obtain the biological processes and molecular functions in which the input gene lists are enriched. The *–log10(FDR)* of the enriched functions were plotted to indicate the significance of the enrichment of each function. Comparisons to existing *Mecp2* mouse gene expression datasets (hypothalamus (66,69), cerebellum (67) and amygdala (68)) were made using lists of altered gene sets as provided in previous studies.

## Gene Expression Omnibus Accession Number

GSE83323

## Supplementary Material

Supplementary Material is available at *HMG* online.

Supplementary Data
