# Supplementary material for: Loss of MeCP2 in the rat models regression, impaired sociability and transcriptional deficits of Rett syndrome
Source: Hum Mol Genet. 2016 Jun 30;25(15):3284–302. doi: 10.1093/hmg/ddw178 (PMC5179927; doi:10.1093/hmg/ddw178)
Supplement: Supplementary Data [file supp_25_15_3284__index.html]

Loss of MeCP2 in the rat models regression, impaired sociability and transcriptional deficits of Rett syndrome — Loss of MeCP2 in the rat models regression, impaired sociability and transcriptional deficits of Rett syndrome — Supplementary Data 

# Loss of MeCP2 in the rat models regression, impaired sociability and transcriptional deficits of Rett syndrome

## Supplementary Data

files

- Supplementary Data - zip file
