# Supplementary material for: Loss of MeCP2 in the rat models regression, impaired sociability and transcriptional deficits of Rett syndrome
Source: Hum Mol Genet. 2016 Jun 30;25(15):3284–302. doi: 10.1093/hmg/ddw178 (PMC5179927; doi:10.1093/hmg/ddw178)
Supplement: Supplementary Data [file supp_ddw178_suppl_data.zip › HMG-2016-TWB-00204_VeeraragavanSM.pdf]

Supplementary Material

Loss of MeCP2 in the rat models regression, impaired sociability and transcriptional deficits of Rett syndrome

Surabi Veeraragavan, Ying-Wooi Wan, Daniel R Connolly, Shannon M Hamilton, Christopher S Ward, Sirena Soriano, Meagan R Pitcher, Christopher M McGraw, Sharon G Huang, Jennie R Green, Lisa A Yuva, Agnes J Liang, Jeffrey L Neul, Dag H Yasui, Janine M LaSalle, Zhandong Liu, Richard Paylor, Rodney C Samaco

Supplementary Materials and Methods with references 2

Supplementary Figure Legends....................4

Supplementary Table Legends....................6

Supplementary Figures....................8

SUPPLEMENTARY MATERIAL

SUPPLEMENTARY MATERIALS AND METHODS

Western blot and immunofluorescence staining. MeCP2 levels were analyzed in additional brain regions as a comparative analysis to cortical tissue; the amygdala and cerebellum were dissected from an additional set of animals independent from the animals in the main text (N=2 per genotype as shown; +/+, female wild-type littermates, and ZFN/+, female *Mecp2*$^{ZFN/+}$ rats) and processed for Western blot analysis using the methods and C-terminal anti-MeCP2 and GAPDH antibodies described in the main text.

For evaluating whether an N-terminal truncation product is detectable, we used male *Mecp2*$^{ZFN/y}$ rats (ZFN/y) compared with male wild-type littermate (+/y) animals (Western blot, N=4 per genotype; IF, N=1 per genotype). IF staining was conducted in parallel with the brain of one *Mecp2* Tm1.1Bird/y mouse, the comparable mouse null allele, and a corresponding male wild-type littermate mouse. All tissue was collected from animals at approximately 6 weeks of life from a separate cohort of the animals in the main text. Cortical tissue was processed using the methods described in the main text, and immunodetection of N-terminal MeCP2 was performed using a commercially available antibody (M7443 diluted 1:1000; Sigma, USA). IF staining was also conducted using the on-slide staining procedure described in the main text; all primary and secondary antibodies as well as DAPI counter-staining were also similar except for the detection of N-terminal MeCP2 (M7443 diluted 1:500; Sigma, USA). Image acquisition was performed using a Zeiss 710 confocal microscope, and prepared with ImageJ as previously described (1).

For quantification of MeCP2 IF signal and percentage relative to NeuN-positive cells, we analyzed the brains from an independent set of female *Mecp2*$^{ZFN/+}$ rats (ZFN/+) and wild-type (+/+) littermate animals (N=3 rats per genotype, approximately 6 weeks of life). Brains were fixed by transcardial perfusion of phosphate buffered saline (PBS) followed by 4% paraformaldehyde (PFA) in PBS. The brains were then removed, placed in PFA overnight, cryoprotected in 30% sucrose in PBS, embedded in optimal cutting temperature medium, and subsequently frozen. Free-floating coronal sections at 50 μm thickness were obtained using a Leica CM3050S cryostat. Matched sections were selected and permeabilized in 0.3% Triton-X 100 in PBS (PBST) for 15 min, and blocked for 2 hr in 10% normal goat serum in PBST (blocking buffer). Sections were then incubated in primary antibody in blocking buffer for 48 hr at 4°C, washed with PBST, incubated in secondary antibody in blocking buffer overnight at 4°C, washed with PBST, incubated in DAPI in

PBST (1:10,000) for 15 min, and washed with PBST. The sections were mounted onto slides using Prolong Gold Antifade Mounting Medium (ThermoFisher Scientific, USA). Primary antibodies used were Chicken anti-MeCP2 (ABE171 diluted 1:500; Millipore, USA) and Mouse anti-NeuN (MAB 377 diluted 1:750; Millipore, USA). Secondary antibodies used included Alexa Fluor conjugated Donkey Anti-Chicken IgG and Donkey Anti-Mouse IgG (1:500; Jackson ImmunoResearch, USA). Slides were imaged using a Zeiss LSM 880 confocal microscope with a 40x objective. Z-stack images were captured with identical image acquisition settings, and the identical region within the somatosensory cortex was imaged using anatomical landmarks based on The Rat Brain in Stereotaxic Coordinates (2). Three sections per animal were imaged and maximum-intensity projections were prepared using ImageJ. Quantitative analysis of MeCP2 intensity in individual neurons was accomplished using Bitplane Imaris 64x version 8.1. Using the Imaris surface tool, surfaces were created to encapsulate individual nuclei based on a threshold DAPI fluorescence intensity and a minimum 3D volume. The mean MeCP2 signal intensity in each nucleus was calculated within surfaces that exceeded a threshold NeuN fluorescence intensity. Surfaces that encapsulated more than 2 nuclei were manually discarded. Quantification of the percentage of NeuN-positive nuclei expressing MeCP2 was performed using a consistent mean MeCP2 intensity threshold. A minimum of 163 cells was quantified per animal from a minimum of 3 images. Statistical analysis was performed as described in the main text using a one-way ANOVA with genotype as the main factor.

SUPPLEMENTARY REFERENCES

1. Ward,C.S., Arvide,E.M., Huang,T.-W., Yoo,J., Noebels,J.L. and Neul,J.L. (2011) MeCP2 is critical within HoxB1-derived tissues of mice for normal lifespan. *J. Neurosci. Off. J. Soc. Neurosci.*, **31**, 10359–10370.

2. Paxinos,G. and Watson,C. (2014) The Rat Brain in Stereotaxic Coordinates Seventh edition. Elsevier/AP, Academic Press is an imprint of Elsevier, Amsterdam ; Boston.

SUPPLEMENTARY FIGURE LEGENDS

Figure S1. Schematic diagram showing the predicted modification of the rat Mecp2 locus using a ZFN approach. **(A)** *Mecp2* contains four exons; ZFN targeting directed towards the fourth exon is predicted to result in an early truncation product that terminates at amino acid 245. The predicted truncation product, though not detected as shown in **Fig. S2**, would have contained a complete methyl-CpG-binding domain (MBD) and a partial transcriptional repression domain (TRD), but lack a nuclear localization signal (NLS).

Figure S2. An N-terminal truncation product is not detected in MeCP2 ZFN rats, similar to observations reported in RTT individuals. (A, B) Analysis of additional brain regions confirm a reduction of MeCP2 in female *Mecp2*$^{ZFN/+}$ rats (ZFN/+) compared with female wild-type littermates (+/+). **(C)** The ZFN-induced deletion is predicted to result in an approximate 27 kD truncation product; however, Western blot analysis using an N-terminal anti-MeCP2 antibody did not detect this product, but rather a non-specific band present in both *Mecp2*$^{ZFN/y}$ (ZFN/y) and male wild-type littermate (+/y) rats. **(D-G)** Combined immunofluorescence staining using both N- and C-terminal anti-MeCP2 antibodies detected MeCP2 in cortical cells of male +/y rats **(D)**. Neither antibody detected MeCP2-specific signal in *Mecp2*$^{ZFN/y}$ rat brain **(E)**. As a control, brains from a male *Mecp2*$^{-/y}$ mouse brain (Tm1.1Bird/y allele, complete null) and wild-type +/y littermate mouse were stained with the same antibodies. Similar to +/y rat brain, +/y mouse brain showed the expected pattern of MeCP2 expression **(F)**. Neither antibody detected specific MeCP2 signal in Tm1.1Bird/y mouse brain **(G)**. The faint signal present using the N-terminal anti-MeCP2 antibody in the rat **(E)** was also present in the Tm1.1Bird/y mouse brain **(F)** confirming the detection of non-specific background signal. These data indicate that the *Mecp2* ZFN rat is a viable model to study the consequences of loss of MeCP2 protein as reported in nonsense mutations associated with RTT that result in loss of protein, no truncation product and normal RNA levels as discussed in the main text. Scale bar indicates 25 μm.

Figure S3. Quantitative analysis of MeCP2 signal intensity and distribution within NeuN-positive (+) cells. (A-C) Representative images of the somatosensory cortex for female wild-type littermates (+/+; **(A)**) and female *Mecp2*$^{ZFN/+}$rats (ZFN/+; **(B)**) are shown. ZFN/+ rats display an approximate 50% reduction in MeCP2 immunofluorescence signal intensity **(C)**. **(D)** Quantification of MeCP2 signal within NeuN-positive (+) cells demonstrates that approximately half of

NeuN+ cells express MeCP2. Although there is some degree of variability among the three animals that were analyzed, the percentage of MeCP2+/NeuN+ cells in ZFN/+ rat somatosensory cortex is significantly reduced among the three animals analyzed. White arrows indicate NeuN+ cells that do not have MeCP2 signal, scale bar indicates 50 µm. +/+, female wild-type littermate; ZFN/+, *Mecp2*$^{ZFN/+}$; * $p < 0.05$.

SUPPLEMENTARY TABLE LEGENDS.

Table ST1. Down common rat-mouse. List of down-regulated gene expression changes in *Mecp2* ZFN/y rat hypothalamus that were "activated" in the hypothalamus of MeCP2 mouse models. +/y, male wild-type littermate; ZFN/y, male *Mecp2*$^{ZFN/y}$ rat.

Table ST2. Up common rat-mouse. List of up-regulated gene expression changes in *Mecp2* ZFN/y rat hypothalamus that were "repressed" in the hypothalamus of MeCP2 mouse models. +/y, male wild-type littermate; ZFN/y, male *Mecp2*$^{ZFN/y}$ rat.

Table ST3. Opposite from *Mecp2*-NULL mice, or similar to *MECP2*-TG mice. List of gene expression changes in *Mecp2* ZFN/y rat hypothalamus that were either opposite from *Mecp2*-NULL mice, or changed in the same direction as observed in *MECP2*-TG mice. +/y, male wild-type littermate; ZFN/y, male *Mecp2*$^{ZFN/y}$ rat.

Table ST4. Opposite from both *Mecp2*-NULL and *MECP2*-TG mice. Gene expression change in *Mecp2* ZFN/y rat hypothalamus that was opposite from both *Mecp2*-NULL and *MECP2*-TG mice. +/y, male wild-type littermate; ZFN/y, male *Mecp2*$^{ZFN/y}$ rat.

Table ST5. Down unique rat. List of gene expression changes uniquely down-regulated in *Mecp2* ZFN/y rat hypothalamus. +/y, male wild-type littermate; ZFN/y, male *Mecp2*$^{ZFN/y}$ rat.

Table ST6. Up unique rat. List of gene expression changes uniquely up-regulated in *Mecp2* ZFN/y rat hypothalamus. +/y, male wild-type littermate; ZFN/y, male *Mecp2*$^{ZFN/y}$ rat.

Table ST7. Down in rat and altered in cerebellum or amygdala of MeCP2 mice. List of gene expression changes down-regulated in *Mecp2* ZFN/y rat hypothalamus and altered in brain regions other than the hypothalamus of MeCP2 mouse models, such as the cerebellum and amygdala. +/y, male wild-type littermate; ZFN/y, male *Mecp2*$^{ZFN/y}$ rat.

Table ST8. Up in rat and altered in cerebellum or amygdala of MeCP2 mice. List of gene expression changes up-regulated in *Mecp2* ZFN/y rat hypothalamus and altered in brain regions other than the hypothalamus of MeCP2 mouse models, such as the cerebellum and amygdala. +/y, male wild-type littermate; ZFN/y, male *Mecp2*$^{ZFN/+}$ rat.

Table ST9. List of gene ontology terms associated with common gene expression alterations. Tables showing list of gene ontology terms associated with down- and up-regulated genes commonly altered among *Mecp2* rats and mice.

Table ST10. List of gene ontology terms associated with unique gene expression alterations. Tables showing list of gene ontology terms associated with down- and up-regulated genes uniquely altered in *Mecp2* rats.

Table ST11. Summary of gene expression findings in RTT brain based on the predictive validity of MeCP2 rodent model transcriptional profiling studies.

Table ST12. Statistical summary of behavioral data. Complete table of statistical analyses for behavioral studies.

Fig S1

A

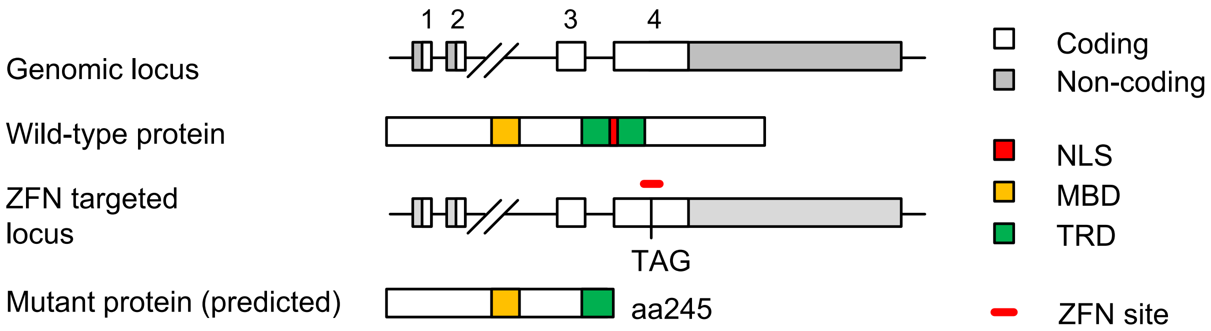

Genomic locus
1
2
3
4
Wild-type protein
ZFN targeted
locus
TAG
Mutant protein (predicted)
aa245
Coding
Non-coding
NLS
MBD
TRD
ZFN site

Fig S2

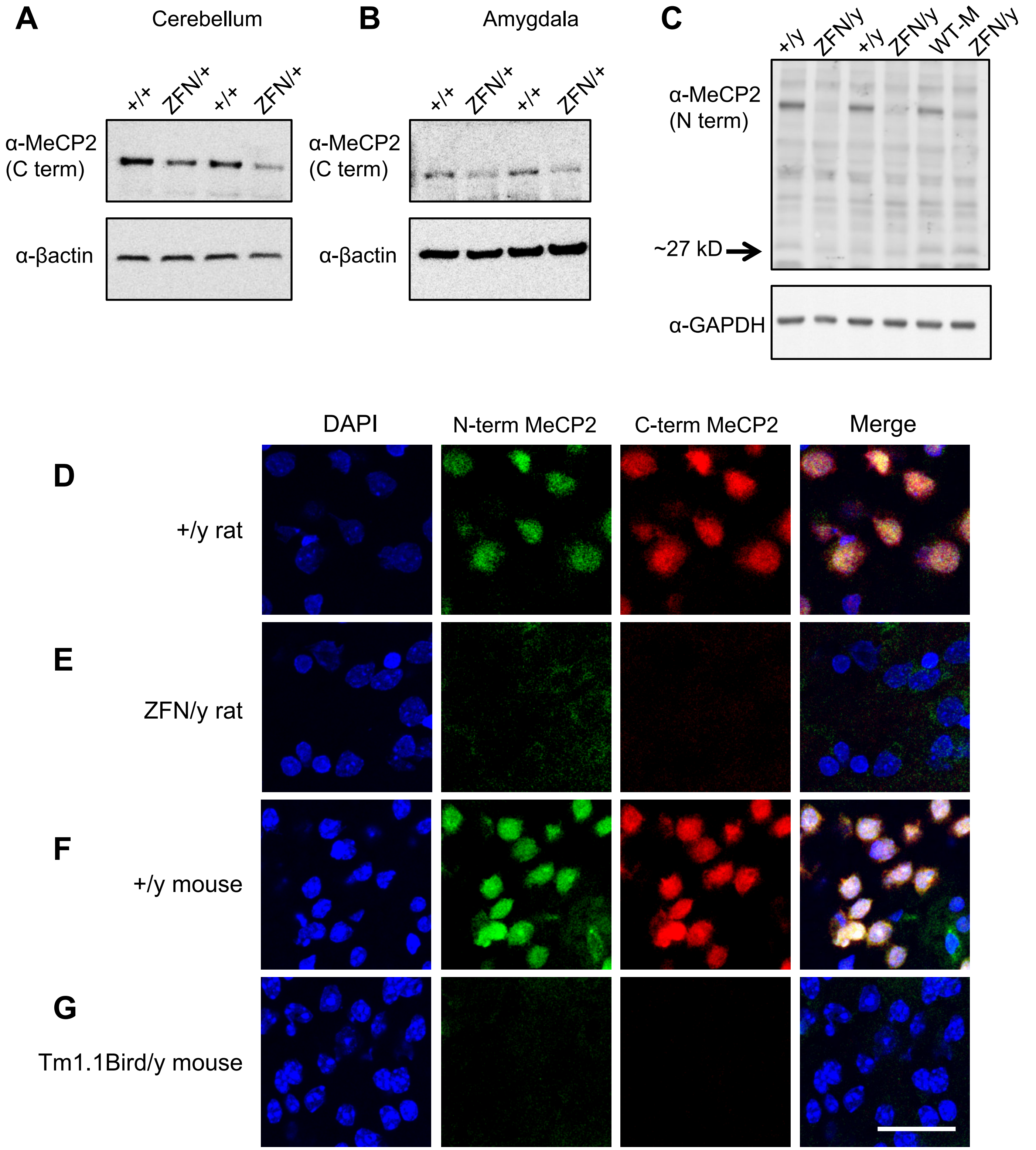
A
Cerebellum
+/+
ZFN/+
+/+
ZFN/+
α-MeCP2
(C term)
α-βactin
B
Amygdala
+/+
ZFN/+
+/+
ZFN/+
α-MeCP2
(C term)
α-βactin
C
+/y
ZFN/y
+/y
ZFN/y
WT-M
ZFN/y
α-MeCP2
(N term)
~27 kD
α-GAPDH
DAPI
N-term MeCP2
C-term MeCP2
Merge
D
+/y rat
E
ZFN/y rat
F
+/y mouse
G
Tm1.1Bird/y mouse

Fig S3

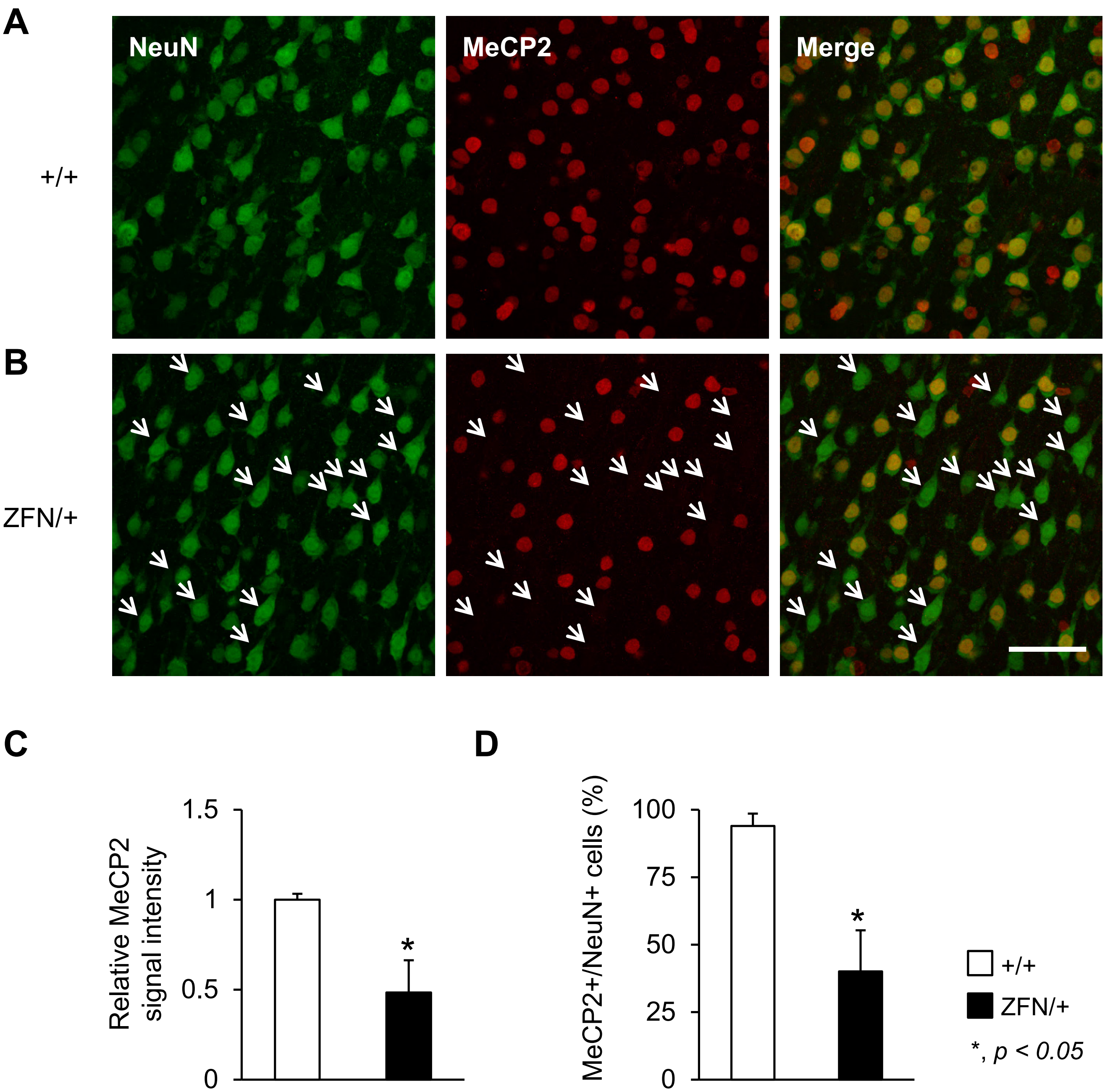
A
B
C
D
NeuN
MeCP2
Merge
+/+
ZFN/+
Relative MeCP2 signal intensity
1.5
1
0.5
0
*
MeCP2+/NeuN+ cells (%)
100
75
50
25
0
*
+/+
ZFN/+
*, p < 0.05
